# Synergistic and Intelligent Hydrogel for Conducting Osteoblast Proliferation: Synthesis, Characterization, and Multifunctional Properties

**DOI:** 10.3390/gels11110910

**Published:** 2025-11-14

**Authors:** Karen Michelle Guillén-Carvajal, Benjamín Valdez-Salas, Ernesto Alonso Beltrán-Partida, Jorge Salomón Salvador-Carlos, Mario Alberto Curiel-Álvarez, Jhonathan Castillo-Saenz, Daniel González-Mendoza, Nelson Cheng

**Affiliations:** 1 Core Facility-Química y Materiales Avanzados, Instituto de Ingeniería, Universidad Autónoma de Baja California, Blvd. Benito Juárez and Normal s/n., Mexicali 21280, Baja California, Mexico; beltrane@uabc.edu.mx (E.A.B.-P.); jsalvador@uabc.edu.mx (J.S.S.-C.); mcuriel@uabc.edu.mx (M.A.C.-Á.); jhonathan.saenz@uabc.edu.mx (J.C.-S.); 2 Instituto de Ciencias Agrícolas, Universidad Autónoma de Baja California, Carretera a Delta s/n C.P. 21705, Ejido Nuevo León, Mexicali 21100, Baja California, Mexico; danielg@uabc.edu.mx; 3 Magna International Pte Ltd., 10 H Enterprise Road, Singapore 629834, Singapore

**Keywords:** smart-hydrogel, osteo-rehabilitation, vitamins, skin-biomaterial, biodegradability

## Abstract

Current trends in intelligent hydrogels design for tissue engineering demand multifunctional biomaterials that respond to external stimuli, while maintaining adhesion, controlled degradation, and cytocompatibility. The present work describes the synthesis and characterization of a novel, intelligent and synergistic hydrogel for promoting osteoblastic growth and regeneration. The hydrogel comprises a complex matrix blend of natural biodegradable polymers, vitamins (A, K2, D3, and E), and bioactive components such as zinc phosphate nanoparticles and manganese-doped hydroxyapatite to improve osteoblastic functionality. The hydrogel proved to have physicochemical properties for recovery and self-healing, highlighting its potential application as an auxiliary in bone rehabilitation. Key parameters such as rheological behavior, moisture content, water absorption, solubility, swelling, biodegradability, and responsiveness to temperature and pH variations were thoroughly evaluated. Furthermore, its adhesion to different surfaces and biocompatibility were confirmed. Skin contact test revealed no inflammatory, allergic, or secondary effects, indicating its safety for medical applications. Importantly, the hydrogel showed high biocompatibility with no cytotoxicity signs, favoring cell migration and highlighting its potential for applications in regenerative medicine.

## 1. Introduction

In recent years, intelligent hydrogels have emerged as one of the most promising strategies for tissue regeneration, particularly in applications related to skin and bone engineering. Their ability to mimic the native extracellular matrix and respond to external stimuli makes them highly attractive for regenerative medicine [1,2]. Recent studies highlight the development of multifunctional hydrogels that not only offer structural support, but also exhibit bioactive properties, self-healing capacity, and adhesiveness to different surfaces, making them key tools for regenerative medicine and wound healing [1,2,3,4,5].

A notable trend in hydrogel design is the synergistic use of both natural and synthetic polymers. This combination leverages the advantages of each type of material while mitigating their individual limitations. For example, natural polymers such as chitosan (CS) and guar gum (GG) can be combined with synthetic polymers like polyvinyl alcohol (PVA) to produce hydrogels with enhanced biocompatibility, mechanical strength, and controlled swelling capacity [6,7,8]. CS, derived from chitin, is well known for its antimicrobial activity, biodegradability, and wound-healing properties, making it a valuable component in biomedical formulations [9,10]. Similarly, gelatin (Gel), derived from collagen (Col), promotes cell adhesion and proliferation, as it is a major component of the extracellular matrix involved in both soft and hard tissues [11,12].

Beyond the polymeric network, the inclusion of vitamins and other bioactive agents further enhances the osteogenic potential of these materials. Interestingly, vitamins such as A, K2, D3, and E serve as cofactors in collagen synthesis, act as antioxidants, and modulate immune responses, thereby accelerating tissue repair and improving integration with the host tissue [13,14]. Likewise, bioactive nanoparticles (NPs) are increasingly being incorporated into hydrogel matrices. For instance, composite hydrogels reinforced with hydroxyapatite or nanoclays exhibit improved mechanical stability, controlled degradation, and enhanced cell–matrix interactions [15,16]. Moreover, recent studies highlight that the incorporation of zinc phosphate nanoparticles (ZnP) and manganese-doped hydroxyapatite (Mn-Ha) not only contributes osteoinductive properties but also regulates osteoclast activity and promotes osseointegration, which are critical for successful bone regeneration [17,18,19,20,21].

Despite these advances, significant challenges remain. Hydrogels often face limitations in achieving the required balance between biodegradability and mechanical stability, which is essential for guiding osteoblast proliferation and new bone formation [22]. Purely polymeric systems typically lack sufficient stiffness to support osteogenesis, while inorganic-particle-reinforced composites may compromise flexibility or biocompatibility [23]. Furthermore, most reported hydrogels incorporate either bioactive polymers or inorganic nanoparticles but rarely integrate a multicomponent design that simultaneously addresses mechanical reinforcement, controlled ion release, osteogenic stimulation, and immunomodulation [24,25]. This lack of integration hampers their translation into clinically relevant scaffolds for bone tissue engineering.

In this study, a multifunctional and intelligent hydrogel was developed, capable of responding to diverse physicochemical and mechanical stimuli. The hydrogel supports osteoblastic proliferation and regeneration and exhibits controlled biodegradability, which is expected to favor the progressive release of ZnP and Mn-Ha under physiological conditions. Rather than serving as a permanent scaffold, this system is conceived as a temporary and bioactive matrix that can assist bone regeneration by providing a supportive microenvironment and delivering functional components. This promising biomaterial therefore holds strong potential for future applications in bone regeneration and regenerative medicine.

## 2. Results and Discussion

### 2.1. Hydrogel Characterization

#### 2.1.1. Synthesis

The proposed interactions within the hydrogel system were inferred from the known reactivity of the components, together with the macroscopic and physicochemical changes observed during preparation (e.g., gelation, color variation, and viscosity). These hypotheses are consistent with mechanisms previously reported for similar hydrogel systems and help explain the structural evolution observed [26]. The following section describes the main interactions hypothesized to occur among the hydrogel components based on these considerations.

The synthesized hydrogels showed different physical crosslinking behavior. First, glucosamine HCl (Glu) helped reduce the pH of the system, providing a suitable environment for chitosan (CS) to protonate. The initial interaction proposes that electrostatic attractions occur between the NH_3_^+^ group with the CS and the carboxylate (-COO^−^) groups of the gel, as well as with the sulfonate (-SO_3_^−^) groups of the chondroitin sulfate (Con) [27]. Guar gum (GG) increased the hydrogel’s apparent viscosity due to water content and hydrogen bonds [8,28]. Moreover, glycerin (Gli) molecules bonded with OH groups of polysaccharides and peptides in collagen (Col), adding plasticity and structure to the matrix molecule. On the other hand, free gelatin (Gel) and Col groups react with GG, and the mechanical behavior varied based on hydrogen bonds and polymer freedom [29]. In this instance, the tetrahydroxyborate ion facilitated secondary cross-linking with GG at the 1,3 diol groups and with Gli. This reaction involved monodiol complexation and borax-diol complex formation, leading to a larger and more defined hydrogel structure. However, the desired structure was still not achieved [28,30,31].

The introduction of tannic acid (TA) enabled partial conjugation with Gel, forming crosslinked aggregates through hydrogen bonding and triple helix complexation. These aggregates were subsequently destabilized via hydrolysis, depolymerization, and pH sensitive TA oxidation. The mild oxidation of catechol groups also promoted covalent interactions with amine groups, further contributing to hydrogel stability [32,33].

The presence of ceramics zinc phosphate (ZnP) and manganese-doped hydroxyapatite (Mn-Ha) particles provided ions (Zn^+2^, Ca^+2^, Mn^+2^ and PO_4_^−3^), within the matrix. These ions formed ionic complexes between the different polysaccharides and peptides in the formulation, improving cohesion and mechanical strength by acting as additional crosslinking sites [34,35]. On the other hand, soy lecithin (Lec) and olivoil avenate (Ol) formed micelles that encapsulated the vitamins, which became suspended within the polymeric network.

The incorporation of polyvinyl alcohol (PVA) further enhanced structural integrity through the formation of borate ester bonds between borate anions and hydroxyl groups on adjacent PVA chains [36]. Notably, Gli facilitated additional hydrogen bonding among PVA chains, while GG stabilized the hydrogel network during the crosslinking process [37]. This strategy enabled the fabrication of structurally controlled hydrogels without the need for energy-intensive methods such as thermal treatment or freeze–thaw cycling [38]. These findings highlight the system’s scalability and precise control over cross-linking kinetics, positioning it as a sustainable platform for tunable hydrogels in drug delivery and tissue engineering.

#### 2.1.2. SEM-EDS

**Figure 1:**
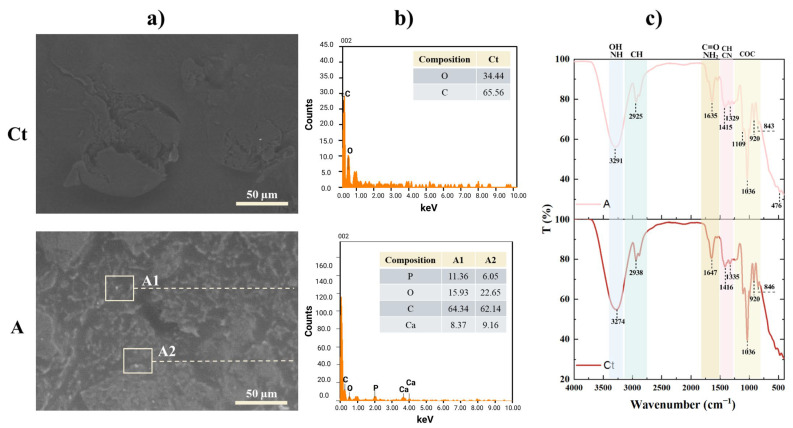
(**a**) SEM micrograph of the evaluated hydrogels, 500× of magnitude; (**b**) EDS analysis of the evaluated hydrogels; (**c**) FTIR spectra of hydrogels Ct and A.

The surface morphology of the hydrogels, as observed via SEM (Figure 1a), revealed distinct structural differences. Hydrogel Ct exhibited a mostly smooth surface with no apparent porosity, indicating a compact matrix. In contrast, hydrogel A displayed a rougher surface with irregular pores averaging 5.88 ± 2.44 µm (Figure S2a), along with a homogeneous distribution of its components [7]. Such porous architecture is generally considered favorable for enhanced swelling behavior and may facilitate the diffusion or potential release of bioactive components. Additionally, nanoparticles with an average of ~205 nm were identified on the surfaces.

The chemical composition of the hydrogels, shown in the EDS analysis (Figure 1b), confirmed the presence of C and O, associated with the polymeric matrix. Moreover, the detected nanoparticles contained P, C and Ca, consistent with the hydroxyapatite incorporated or formed within the structure, and correlating with the observed particle sizes. In addition, the XRF analysis shown in Figure S3 confirms the presence of P, Ca, Mn, Zn, S, and K in hydrogel A. 

#### 2.1.3. FTIR Analysis

The FTIR spectra of the hydrogels were analyzed (Figure 1c). Hydrogel Ct and A exhibited similar spectra after drying. The peaks at 3291 cm^−1^ and 3274 cm^−1^ indicate O-H stretching vibrations. These peaks are not as broad as those shown by the GG spectrum according to the literature [39], due to the consumption of hydroxyl groups in the formation of covalent bonds with Bx [30], in addition to other elements of the hydrogel such as PVA. The higher the amount of Bx, the less prominent the O-H stretching frequency becomes [28]. Likewise, AT contributes a broad band by phenolic O-H stretching due to hydrogen bonds [40].

C-H stretching bands were found at 2938 cm^−1^ (Ct) and 2925 cm^−1^ (A). The bands at 1647 cm^−1^ and 1635 cm^−1^ are assigned to the O-H bending modes of the functional groups from GG, PVA, and Gli; as well as to the carboxylic acid (-C=O) groups and δ (-NH_2_) Gel, Col, and CS, in a minor presence. Additionally, they correspond to the formation of hydrogen bonds between chains of GG, Gli, Gel, and Col with PVA or between them, suggesting internal crosslinking [41], and to the stretching of the ring structure of mannose in GG [42]. The bands near 1415 cm^−1^ correspond to -C-H and -C-N bending vibrations. Several characteristic peaks of PVA (approximately 1750 cm^−1^, 1250 cm^−1^, and 1120 cm^−1^) disappeared or were drastically reduced in both samples, indicating miscibility, bond formation, and complete mixing with the rest of the actives [7].

It is important to note that the absence of the band at 1715 cm^−1^, characteristic of AT, may be due to the increased interaction and bond formation as a strong indication of the successful integration of the polymers. In the study presented by Abouelmagd et al. [40], it was observed that when this band was present in a mixture of AT and gatifloxacin, instead of decreasing, the intensity of this band increased, which confirmed the weak interaction between these compounds. Therefore, we can suggest that in our hydrogel, these elements are fully integrated and promote stronger interactions.

On the other hand, in the hydrogels, the -C-O stretching band appears at 1036 cm^−1^ [43]. The characteristic Gli bands are located in the region from 800 cm^−1^ to 1150 cm^−1^ and correspond to the vibrations of -C-C and -C-O bonds. The peaks between 840 cm^−1^ and 925 cm^−1^ are assigned to -C-C skeletal vibrations. Finally, the peak at 476 cm^−1^ corresponds to the stretching frequency of Zn and Mn present in hydrogel A [44].

Since no noticeable differences were observed between the spectra of the hydrogels, it is confirmed that the active components have been incorporated into the polymeric matrix and are generally distributed in the heteropolymer cross-linking. Therefore, the polymeric base components diminished the peaks of the other components, indicating their dominant presence, as expected. This may be since the hydrogels share the same polymeric base and yet exhibit different characteristics, thus suggesting the presence and influence of additional elements.

#### 2.1.4. AFM

**Figure 2:**
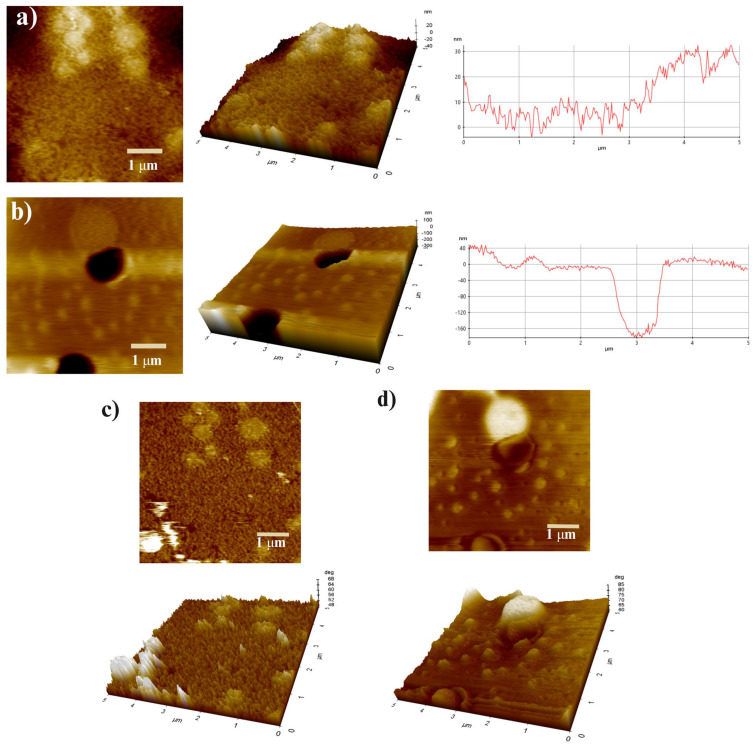
(**a**) AFM topographic analysis of Control (Ct) and (**b**) hydrogel A. The images are presented in both 2D and 3D orientations, together with the height profile of the samples (arranged from left to right). (**c**) Phase study of Control (Ct) and (**d**) hydrogel A. The images are shown in both 2D and 3D orientations (**top** and **bottom**).

The topography of the hydrogels was evaluated by AFM (Figure 2). The surface of hydrogel Ct (Figure 2a) was much rougher than that of hydrogel A, with a maximum height of 32.31 nm. This type of topography allows greater adhesion to various surfaces, consistent with the adhesion test data, as the Ct hydrogel showed better adhesion to all surfaces tested.

Our research has highlighted the unique properties of Hydrogel A (Figure 2b), which shows evenly distributed, spherical protrusions, thus suggesting that the micro- and nanoparticles are embedded in the polymeric matrix. The sizes obtained by SEM for ZnP, Mn-Ha, and Ha (Supplementary Materials, Figures S2b, S4 and S5) indicate that these bioactives are within the size range (ZnP, length 1.76 µm and width 0.202 µm; Mn-Ha, diameter ~ 90 nm; Ha, diameter ~ 254 nm). On the other hand, hydrogel A exhibited lower porosity (diameter size ~ 0.950 µm), resulting in higher roughness (Ra = 42.534 nm), as confirmed by SEM micrograph analysis of the hydrogels. These surface properties play a crucial role in adhesion to various surfaces and in biological contexts, influencing cell adhesion, migration, and proliferation [45]. Experimental studies have shown that micro-/nano surface topographies, such as porous and rough structures, promote mechanical anchoring of mammalian cells, facilitate the exchange of ions and signaling molecules, and enhance nutrient transport, generating a more stable microenvironment for cell growth [46].

Figure 2c,d shows the phase images of both Ct and A hydrogels. In hydrogel Ct, a small portion of not-fully-integrated material was observed [47]. In contrast, in hydrogel A, a distinct difference between the polymeric surface and the embedded nanoparticles (NPs) was observed, making it evident that the NPs had been coated by the same components of the polymeric matrix. This resulted in a lower contrast in the images, suggesting reduced variability in the topographic homogeneity of the material. In addition, the efficient mixing achieved during the fabrication process was verified, confirming the better integration of the components in hydrogel A.

#### 2.1.5. Humidity Content

**Figure 3:**
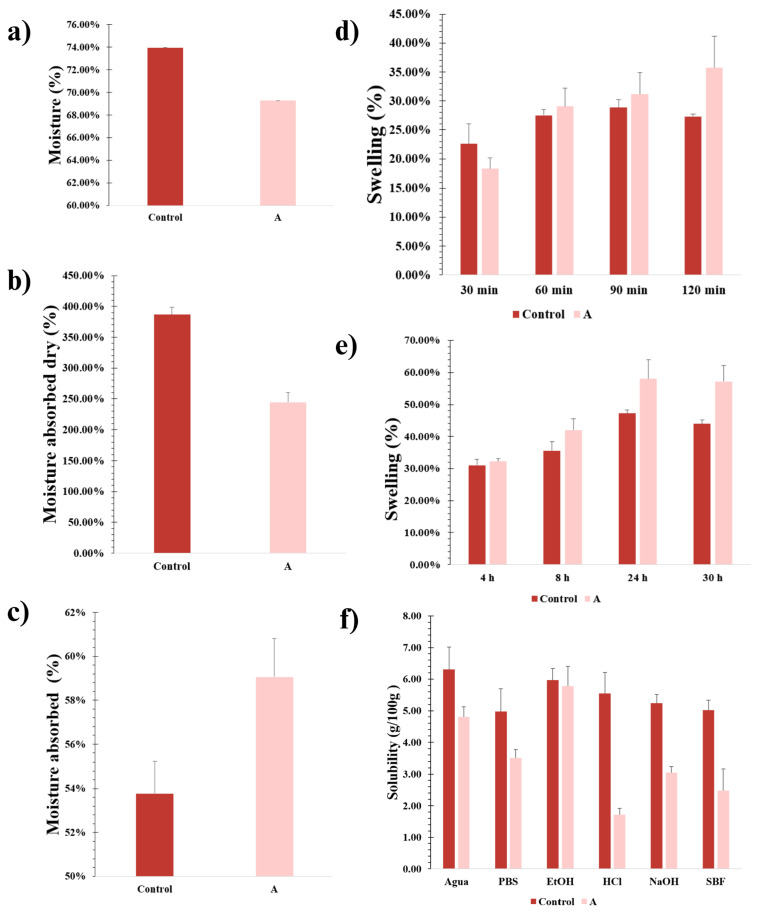
(**a**) Analysis of the amount of moisture contained in each hydrogel; (**b**) Water absorption ability from the studied hydrogels while dry; (**c**) Water absorption ability from the studied hydrogels in normal conditions; (**d**) Hydrogel swelling ability using a PBS solution during 30 min intervals; (**d**,**e**) Hydrogel swelling ability using a PBS solution during longer time periods; (**f**) hydrogel solubility in water, PBS, EtOH, HCl, NaOH and SBF. Data are presented as mean ± SD, *n* = 3.

The samples were stored in a desiccator for 5 days to determine the moisture content. According to Figure 3a, hydrogel Ct presented a higher value in its moisture content of 73.94%, compared to hydrogel A, which had 69.27%. Therefore, the polymeric matrix of each hydrogel represented 26.06% (Ct) and 30.73% (A) of the total mass. The high water content in hydrogels enhances permeability and porosity, allowing rapid diffusion of oxygen and nutrients, which promotes cell growth [48,49].

#### 2.1.6. Absorbed Humidity

Two tests were performed to determine the amount of water absorbed: one under dry conditions and another under normal conditions (Figure 3b,c). Hydrogel Ct absorbed 378% while dry and 54% under normal conditions before losing structural integrity due to excessive expansion, increased osmotic pressure, and decreased attractive forces [28]. Hydrogel A maintained its structure, indicating better crosslinking, with absorption values of 245% (dry) and 59% (normal conditions), demonstrating superior crosslinking. The high absorption capacity of hydrogel A was primarily attributed to the free functional groups, which play a crucial role in interacting with the water molecules present in Gli and PVA, and to the reinforcement of the polymeric network provided by the other components [50].

#### 2.1.7. Swelling

The swelling behavior of the hydrogels was evaluated by measuring PBS absorption every 30 min over a 2 h period. As shown in Figure 3d, hydrogel A did not reach its maximum swelling within this time frame, achieving >35.75% swelling at 2 h and continuing to expand thereafter. In contrast, hydrogel Ct reached its maximum swelling (28.88%) between 90 and 120 min, followed by a slight decrease in the final measurement.

Extended swelling tests were performed by leaving the hydrogels undisturbed for longer periods (Figure 3e). In this evaluation, both hydrogels displayed a near-linear increase in swelling percentage for up to 24 h, followed by a decline. This behavior is particularly favorable, as the first 24 h post-application are critical for the uptake of nutrients, growth factors, and signaling molecules necessary for cell adhesion, proliferation, and differentiation. Among the two, hydrogel A demonstrated the highest swelling (57.96%) compared to hydrogel Ct (47.23%). Between 18 and 24 h, hydrogel A continued to swell at a rate of 6.17%, followed by a slight reduction of 0.78% between 24 and 30 h. These results suggest that hydrogel A responds more dynamically and controllably to its environment than hydrogel Ct.

The ability of hydrogels to increase their volume is essential, as it facilitates the retention and incorporation of higher concentrations of solutes, biomolecules, and growth factors in the target area for cell stimulation. This property favors the generation of a suitable microenvironment, enhancing both cell migration and bone tissue maturation [51].

In addition, the increase in volume is generally associated with the swelling capacity of hydrogels, which can enable a controlled and sustained release of bioactive agents, such as growth factors and signaling molecules that promote cell growth and differentiation. This property is particularly relevant in tissue engineering applications, as it supports cell proliferation, osteogenic differentiation, and integration of implants with host tissue. Therefore, optimizing the swelling behavior of hydrogels is considered a key factor for successful bone regeneration.

### 2.2. Rheological Studies of Hydrogels

#### 2.2.1. Frequency Sweep

**Figure 4:**
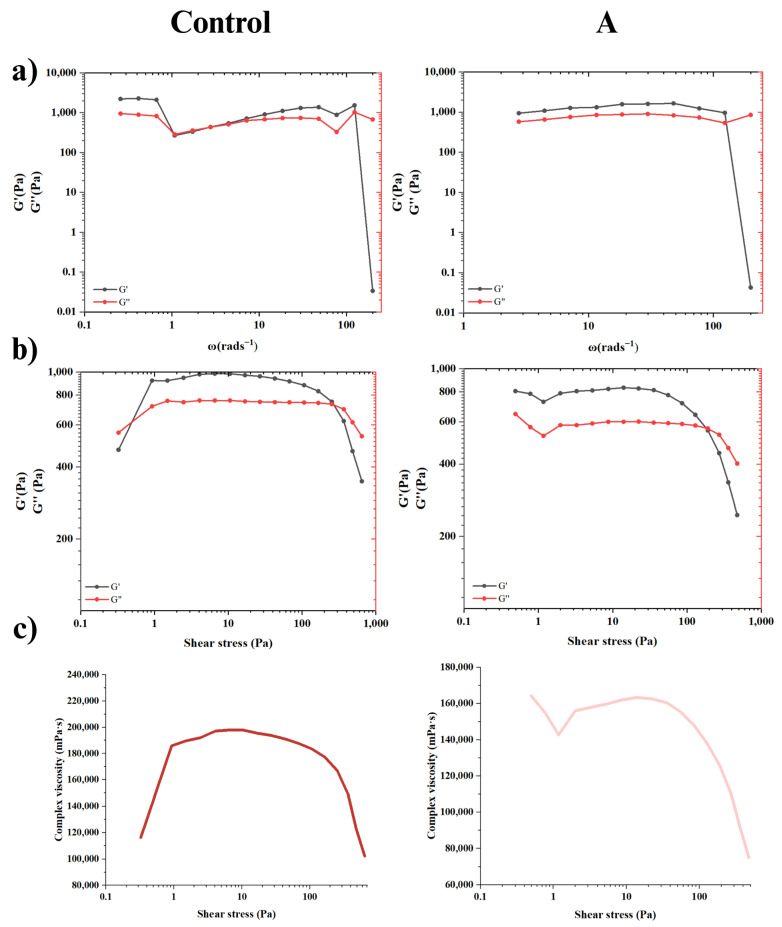
Rheological tests of the developed hydrogels. (**a**) Storage Modulus (G′)/Loss Modulus (G″) vs. Ct and A frequency; (**b**) G′/G″ vs. sheer force of Ct and A; (**c**) complex viscosity vs. sheer stress of Ct and A.

The rheological behavior of the hydrogels was evaluated and is presented in Figure 4. Frequency sweep results (Figure 4a) show that Ct hydrogel was the only one that presented two crossover points between its moduli, where G″ > G′, indicating a predominance of viscous (liquid-like) behavior at low frequency (1.08 rad s^−1^) and high frequency (124 rad s^−1^). The fact that G″ was slightly higher at lower frequencies suggests polymer-chain relaxation and/or weak physical interactions, implying that the Ct hydrogel behaved as a more fluid system. In addition, there was no significant separation between the moduli, which reduces the stability of the material, as it can easily alternate between elastic and viscous behavior. On the other hand, the presence of fluctuations during the test suggests structural heterogeneity or the formation of aggregates within the polymeric network [52]. In contrast, hydrogel A showed an independent behavior over a wider frequency range, indicating that it has a stable and well-crosslinked polymeric network. Similarly, since G′ > G″, we can state that the material behaves as an elastic solid and the point of the solid-to-liquid transition occurred at higher frequencies (124 rad s^−1^) with a modulus value of 563.18 Pa. The constancy of G′, without a significant separation from G″, suggests the coexistence of both covalent and physical crosslinking, the latter predominating. The presence of a crossover point also indicated reversible crosslinking, which is characteristic of self-healing behavior [53].

#### 2.2.2. Deformation Sweep

In Figure 4b, the responses of hydrogels to increasing deformation are shown, with specific focus on mechanical resistance, structural stability, and the deformation limits of the biomaterials. Notably, the Ct hydrogel exhibited two distinct crossover points between the storage and loss moduli. The first crossover occurred at a low shear stress (0.49 Pa). Immediately after, G’ increased, indicating a change in behavior to an elastic solid, thus demonstrating its behavior as a non-Newtonian fluid [54].

On the other hand, hydrogel A also exhibited a decrease in both moduli under increasing stress; however, it did not reach a crossover point, suggesting a more stable and cohesive network under deformation. The linear viscoelastic region (LVR) was observed in both hydrogels at low shear stress levels, where G′ and G″ remained nearly constant despite the increasing strain. This behavior reflects structural stability and predictable mechanical performance within the LVR range [53,54].

Maximum shear stress values of 186 Pa (Ct) and 41.8 Pa (A) were obtained. In general terms, the G’ values indicated a stable and flexible structure with both physical and covalent crosslinking, as mentioned before, mainly in hydrogel Ct. However, within the LVR, the microstructure of the hydrogels did not suffer irreversible damage; beyond these limits, the polymer underwent deformation. The hydrogels displayed a gradual decrease in modulus value outside the LVR, suggesting a reorganization or self-repair of the structure, confirming their dynamic behavior. In the case of A, it was more susceptible to deformations [55]. Finally, the yield stress point (γ) obtained in each hydrogel was 1228.92 Pa (Ct) and 564.77 Pa (A). In general, the Ct hydrogel exhibited a stiffer polymeric network than hydrogel A, due to the absence of the bioactive additives, which have a significant impact on the hydrogel structure [56].

#### 2.2.3. Viscosity and Behavior Identification

Figure 4c presents the complex viscosity ($\dot{\eta }$) of the hydrogels as a function of shear stress, providing key insights into their mechanical responses under deformation. Hydrogel Ct exhibited the highest peak viscosity, reaching 197,920 cP, while hydrogel A showed a slightly lower value of 163,380 cP. Both materials displayed similar overall trends in their flow curves, with a general decrease in viscosity as shear stress increased.

At low shear stress, hydrogel Ct exhibited an initial increase in viscosity, characteristic of shear-thickening (dilatant) behavior [57]. Afterward, a relatively abrupt decline in viscosity was observed at higher shear stresses, indicating partial network disruption. In contrast, hydrogel A displayed a more gradual viscosity response, with a slight initial decrease, followed by a shear-thickening phase, and eventually transitioning to shear-thinning behavior at higher shear rates.

At high deformation, both hydrogels exhibited pseudoplastic behavior, where viscosity decreased as shear stress increased. This shear-thinning effect is typically associated with the disentanglement of polymer chains and the temporary disruption of physical crosslinks, allowing the material to flow more easily [57,58]. In the case of hydrogel A, the smoother viscosity decline suggests a structure dominated by physical interactions, such as hydrogen bonding and electrostatic attractions, rather than permanent covalent crosslinking. These observations were further supported by flow curves of both hydrogels (Figure S6a,b). Such rheological behavior is consistent with a crosslinked, self-healing hydrogel capable of recovering after deformation.

These rheological profiles have significant implications for drug delivery and bone tissue engineering applications. Materials with shear-thinning properties can reduce flow resistance during injection or applications, allowing better moldability within irregular bone defects, while recovering viscosity post-deformation to retain structural integrity. The dominance of reversible physical interactions in hydrogel A, along with its controlled viscosity behavior, suggests its suitability for sustained and localized therapeutic release. These findings highlight the practical relevance and functional versatility of the developed hydrogels for biomedical and pharmaceutical contexts.

### 2.3. Hydrogel Characterization—External Stimuli

#### 2.3.1. Solubility

**Figure 5:**
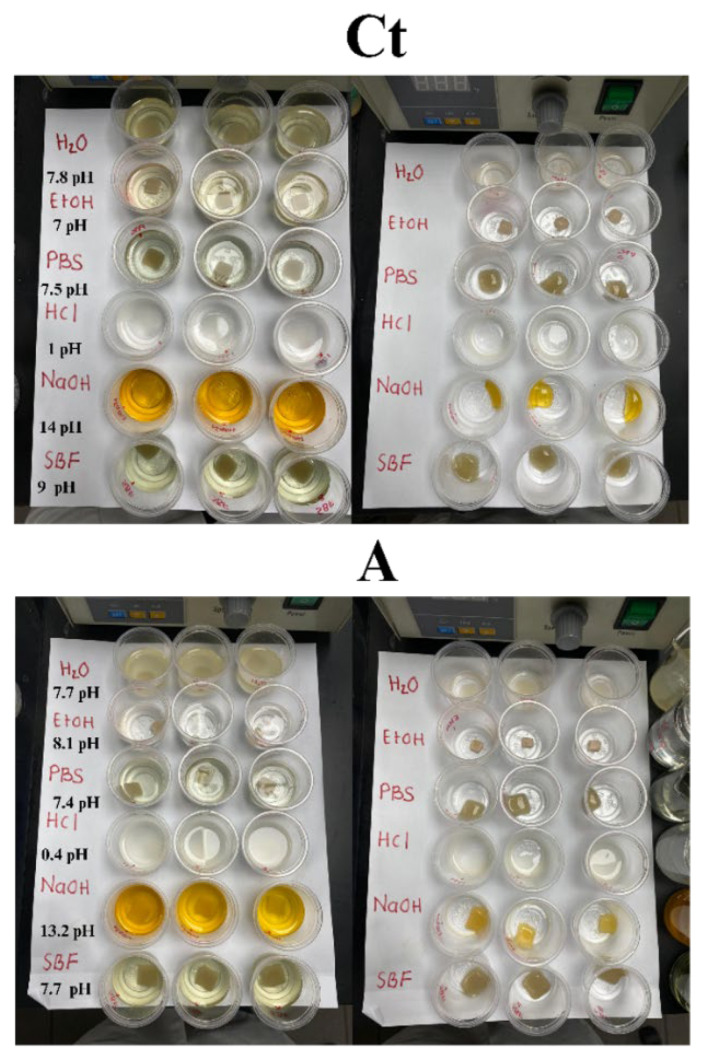
Hydrogel appearance after being exposed for 24 h to different media, as well as when the medium was removed.

The solubility and swelling behaviors of hydrogels are highly dependent on the chemical composition of the surrounding medium. To evaluate this, hydrogel samples were immersed in 10 mL of various solvents for 24 h (distilled water, PBS, a 1 N HCl solution, a 1 N NaOH solution, a 70% (*wt*/*v*) EtOH, and SBF) (Figure 5). Qualitative comparisons with the swelling tests revealed consistent trends. Hydrogel Ct lost its structural integrity and shape in most media, suggesting partial or complete dissolution. In contrast, hydrogel A maintained its shape and cohesion under almost all tested conditions, with superior performance in PBS and SBF, both highly relevant to biomedical applications. Notably, the pH of PBS and SBF increased significantly in the presence of hydrogel Ct, reaching 7.5 and 9.0, respectively, which could be attributed to the partial release of basic components or degradation by-products. In comparison, hydrogel A maintained the physiological pH of PBS (7.4) and caused only a slight increase in the SBF medium to pH 7.7, suggesting improved chemical stability and compatibility under physiological-like conditions.

In highly acidic media, the crosslinked network of the hydrogels exhibited minimal ionization, leading to structural collapse due to dissolution. Since the hydrogel matrix is predominantly stabilized by electrostatic interactions, a sharp decrease in pH caused protonation of amine and carboxylate groups, increasing the net positive charge throughout the network. This disrupted ionic bonds, enhanced hydrophilicity, and contributed to the breakdown of the polymeric matrix [59,60].

Conversely, in alkaline conditions, tannic acid was released and oxidized during the initial stages of exposure, forming new bonds with amino groups and sodium ions within the polymeric network. This reaction promoted the compaction of the hydrogel matrix, improving structural integrity under basic conditions [32,61].

In ethanol, solubility behavior was significantly influenced by its miscibility with water, which enhanced hydrogen bonding within the hydrogel. Ethanol interacted with polar groups in Gel and Col, displacing water and inducing aggregation of these molecules [62]. Notably, as a non-solvent for Gel, ethanol promoted charge neutralization, facilitating the unfolding of Gel chains and exposing aromatic amino acids such as tryptophan, tyrosine, and phenylalanine [63]. These exposed hydrophobic regions reinforced internal hydrophobic interactions, while hydrophilic groups reorganized and established stronger internal hydrogen bonds [64]. As a result, ethanol not only dehydrated but also structurally modified Gel, enhancing intra-network cohesion [65].

Additionally, anions such as HPO_4_^−2^, NH_4_^+^ and SO_4_^−2^, present in the hydrogel and according to the Hofmeister series, could induce a “salification” effect, promoting aggregation, unfolding, and precipitation of gelatin, thereby preserving its native conformation and improving the stability of the proteins contained in the hydrogel [66,67]. This compaction confers a more rigid structure. Comparatively, Peng et al. [68] demonstrated that gelatin precipitation with ethanol and ammonium sulfate produces a compact, smooth gelatin film, where the Hofmeister effect improves the mechanical properties of this film due to the association of hydrogen bonds and hydrophobic interaction. Quantitatively, higher solubility in the EtOH medium was observed in Figure 3f, consistent with the loss of water and other hydrophilic components, as well as the compaction of the polymeric networks.

In general, hydrogel Ct exhibited greater solubility in all tested media, whereas hydrogel A maintained a lower solubility profile, ensuring greater structural stability and suggesting potential for sustained component retention. Only in the HCl medium did the hydrogels partially lose their physical and cohesive form due to the disruption of the bonds responsible for the main crosslinking, while other interactions remained intact, thus preventing complete solubilization of their components.

Considering the practicality of hydrogels, there are significant advantages to low solubility, or in our case, mostly controlled solubility, as it ensures that hydrogels maintain their structure in physiological media for a prolonged period, potentially allowing for a controlled and sustained release of active components. Moreover, hydrogels with lower solubility degrade more slowly, releasing fewer by-products into the physiological environment and thereby reducing the risk of toxicity or adverse reactions. In comparison with the biodegradability results, it can be inferred that our hydrogels remain stable for an extended period, while gradually disintegrating in the medium and potentially responding to environmental stimuli.

#### 2.3.2. Biodegradability

**Figure 6:**
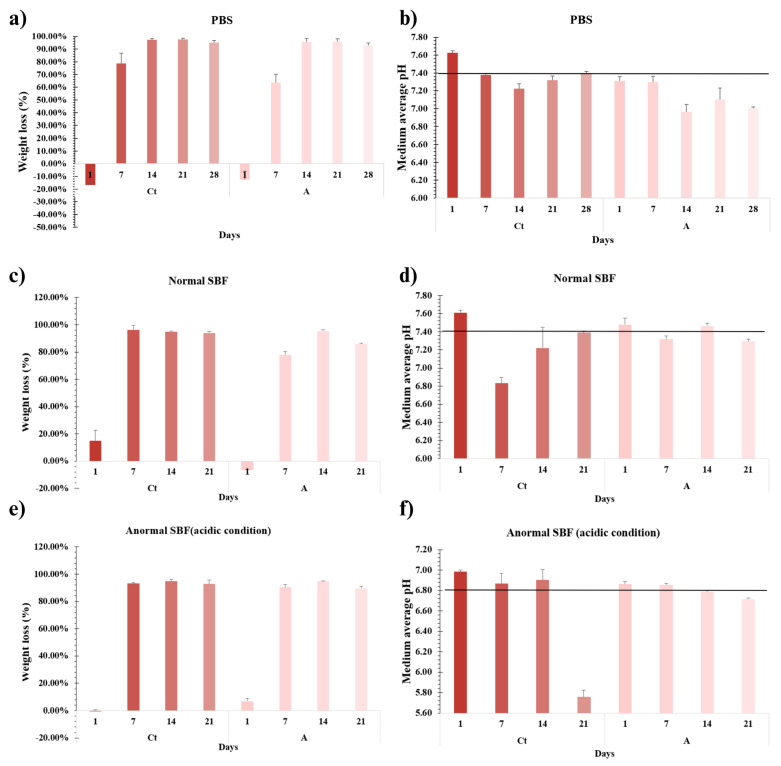
(**a**) Biodegradability linked to weight loss in PBS medium performed on Ct and A hydrogels at 7-day intervals for 28 days; (**b**) pH of the PBS medium during the biodegradability test. The black line indicates the initial pH of the PBS medium; (**c**,**e**) weight loss in both normal and abnormal (pH 6.8) SBF medium of Ct and A hydrogels at 7-day intervals over 21 days; (**d**,**f**) pH of normal and abnormal SBF medium during the biodegradability test performed on the hydrogels. The black line refers to the initial pH of the SBF medium. Data are presented as mean ± SD, *n* = 3.

Biodegradability tests were conducted under controlled conditions for 1, 7, 14, 21, and 28 days (Figure 6a). On the first day, the hydrogels showed swelling without evidence of biodegradation. This characteristic is particularly promising for the developed polymeric systems, as it indicates their ability to absorb the surrounding medium while maintaining structural integrity, facilitating diffusion and exchange of active compounds.

On the other hand, mass loss was first detected after the initial medium change (day 3), becoming more pronounced by day 7. The hydrogels reached a maximum biodegradability of over 95% by day 14, after which a slight decrease was observed at days 21 and 28, likely due to the formation of insoluble by-products (precipitated solids).

The appearance of brown deposits within the hydrogel matrix or at the bottom of the container may be attributed to the precipitation of insoluble complexes or specific chemical interactions between hydrogel components and the medium. One plausible explanation is the formation of tannate complexes through the reaction of tannic acid with metal ions [69]. As the hydrogels have two important NPs (ZnP and Mn-Ha), interactions between these two compounds are possible, either by releasing Mn^+2^ and Zn^+2^ ions into the medium or by reacting directly with them, forming such insoluble complexes [70]. On the other hand, AT is susceptible to oxidation in the presence of oxygen, light, or metal ions. Conditions such as neutral or basic pH accelerate the oxidation of polyphenols, which occur in both PBS and SBF. Among the oxidation products are quinones and phenolic polymers, which tend to become insoluble and develop a brownish color [71,72].

The hydrogels had a noticeable effect on the pH of the medium, which plays a crucial role in maintaining optimal cellular conditions. The pH of the PBS buffer is approximately 7.4, where cellular activity is optimal. An imbalance in pH of the medium can have significant implications for cell viability and indicate ongoing processes within the medium. As can be seen in Figure 6b, the pH of the medium decreased over time from day 1 to day 14, with the most important pH drop in each hydrogel occurring on day 14. After this period, the pH of the media began to increase.

#### 2.3.3. Behavior in Normal and Abnormal SBF

The behavior observed in SBF under both normal and mildly acidic conditions (pH 6.8) is shown in Figure 6c and 6e as well as Figure 6d and 6f, respectively. For hydrogel A, the initial pH was high and close to that of the standard SBF on day 1. After 7 days, the pH of the medium slightly decreased in standard SBF, followed by a gradual recovery toward its initial value after 14 days. By day 21, a slight decrease was observed again. In contrast, under acidic SBF conditions, the pH progressively declined over time. Although these changes were not statistically significant for hydrogel A, this behavior suggests that the material may exhibit a buffering capacity that helps maintain the medium near neutrality, possibly associated with a gradual interaction or partial dissolution of its components.

In both media, hydrogel Ct exhibited a different trend. The pH of the medium initially increased by ~0.2 units, followed by a significant decrease (0.6 units) after 7 days in standard SBF. Subsequently, the pH rose to 7.2 after 14 days and stabilized around 7.4 by day 21. This behavior could be associated with hydrogel mass loss during immersion. A minimal mass loss was recorded on day 1, sufficient to slightly raise the medium’s pH. After 7 days, degradation increased substantially, accelerating hydrogel-medium interactions and resulting in a pH decrease. After another 7 days, the medium began to stabilize, and the pH increased again. A similar pattern was observed in SBF A (acidic SBF), although the change between days 14 and 21 was more abrupt (pH 6.9 to 5.76).

It is well established that the microenvironment in wounds and during bone resorption tends to be acidic (pH 6.69–6.89) [73,74], which hinders healing and osseointegration due to osteoclastic activity. However, the initial pH increase observed with the hydrogels during the first days of testing may promote conditions closer to neutrality, favoring osteoblastic activity and the onset of regeneration. Over time, osteocytes and mineral compounds such as hydroxyapatite are formed, after which the pH of the medium gradually decreases again, resuming the normal bone remodeling cycle [75]. The balance between osteoblasts and osteoclasts is tightly regulated and essential for maintaining bone homeostasis [76].

Likewise, degradation levels above 95% were observed, similar to those present in PBS, where the hydrogel was no longer visible, but precipitated material that had previously been observed embedded in the hydrogel was found. In the formation of apatite or related species, the amount of dissolved phosphate in the media decreases, consuming mainly HPO_4_^−2^ or PO_4_^−3^, causing the equilibrium of the system to shift to compensate for the decrease in these ions, releasing H^+^ to the medium, which in parallel decreases the pH. In the work of Haibo and Mei, they carried out the study of apatite formation at different pH levels, as well as at three different temperatures. Among their results, they noted the correlation between a greater formation of apatite as the temperature increased, with an initial increase in pH and then a decrease due to apatite deposition. Likewise, if the initial pH is neutral or slightly alkaline, the apatite layer formed is denser than when the pH is low, where this layer is more porous. At a temperature of 40 °C, close to body temperature, carbonate apatite, present in bones, is synthesized regardless of the initial pH [77].

During the formation of apatite, several concurrent reactions take place. One involves the decomposition of bicarbonate ions (1), while another corresponds to the interaction between Ca^2+^ and HPO_4_^2−^ ions, leading to the nucleation of apatite on the substrate surface or within the SBF solution. The nuclei that form on the substrate progressively develops a coherent apatite coating, whereas those formed in solution aggregate into precipitates. As the apatite layer or precipitates continue to grow, the pH of the medium gradually decreases due to the consumption of OH^−^ ions (2) [78,79]. On the other hand, octacalcium phosphate (OCP), which is a precursor of hydroxyapatite, can be formed according to reaction (3), where the generation of H^+^ in the medium is observed [79].

HCO_3_^−^ → CO_2_ + OH^−^ (1)

5Ca_2_^+2^ y 3HPO_4_^−2^ + 4OH^−^ → Ca_5_(PO_4_)_3_OH + 3H_2_O (2)

8Ca_2_^+2^ y 6HPO_4_^−2^ + 5H_2_O → Ca_8_H_2_(PO_4_)_6_∙5H_2_O + 4H^+^ (3)

The reduction in pH in the medium observed in our tests may be attributed to the formation of apatite or related calcium phosphate species, which can eventually transform into hydroxyapatite, thereby enhancing the osteoactive ability of our hydrogels.

#### 2.3.4. Self-Healing and Self-Recovery Abilities of Hydrogel

**Figure 7:**
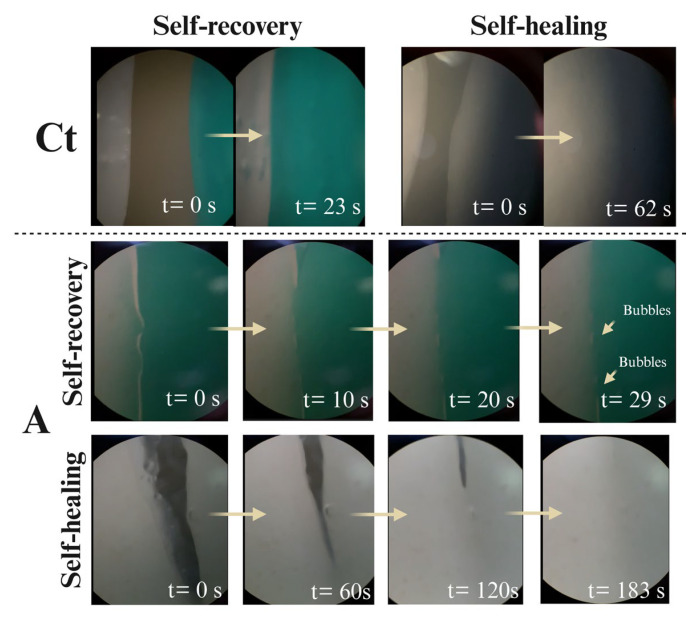
Optical micrographs showing evidence of recovery of contact and non-contact capacity in two separate pieces of hydrogel during the self-recovery and self-healing tests (*n* = 3).

The recovery ability was determined by placing two hydrogel pieces side by side and applying a displacement force so that they would touch. The two pieces were joined to form a single piece, achieving instantaneous adhesion and recovery in <23 s (Ct) and <29 s (A) (Figure 7). Adhesion was confirmed by moving the junction in opposite directions, showing no separation. The recovery capacity was determined by longitudinally cutting a piece of the hydrogel in two parts and recording the time required for reattached. Hydrogel Ct had a closure rate of 3.5 × 10^−3^ cm^2^ s^−1^ while A showed 4.79 × 10^−4^ cm^2^ s^−1^. Although A healed seven times slower than Ct, this may indicate greater cohesion and permanence of the new form taken.

Therefore, our results suggest an outstanding self-healing capability, as the self-recovery rate of hydrogels crosslinked with borax, PVA, and TA varies in the order of minutes (>5 min) [39,80,81]. The recovery characteristics demonstrated by the hydrogels are explained by the dynamic and versatile nature of the diol complexes (O-B-O), which can be easily broken, reformed, and restored by hydrogen bonds provided by PVA, Gli, and TA, and a slight ionic interaction given by ZnP and Mn-Ha in hydrogel A [82]. Meanwhile, Gel, Col, and GG from Schiff-base bonds between their amino and carbonyl groups, producing a nitrogen-carbon double bond. Moreover, chain mobility and the presence of borate ions facilitate the formation of internal bonds across the contact interface [83,84,85]. These findings not only demonstrate the potential use of our hydrogels for biomedical applications but also suggest a remarkable suitability for the fabrication of smart devices, with a high capacity for self-recovery and response to stimuli such as temperature and pH [86,87].

#### 2.3.5. Behavior of the Hydrogel at Different Temperatures

The Ct hydrogel flowed at 38 °C, while A flowed at 41 °C. This behavior is attributed to the incorporation of multiple active ingredients, which improved the crosslinked network, providing semi-crystalline areas within the polymer matrix and thereby increasing its ability to maintain structural integrity at higher temperatures. These values allow the hydrogels to remain gelled temporarily at 37 °C, while other types of processes are performed on them, such as biodegradability and the formation of important species with the physiological medium, as observed in biodegradability tests in PBS and SBF. This similarity to body temperature is mainly attributed to the formation of physical bonds established by Gel and Col, indicating a significant contribution of these compounds to the main polymer network [10,63,88]. These types of bonds fall under reversible bonds, since once the hydrogel returns to a lower temperature, it recovers its structure and reforms the gel. This behavior is also consistent with rheological analyses, which reflect the temperature-dependent viscoelastic response of the system.

In contrast, the hydrogels withstood subzero temperatures without freezing, down to −15.5 °C (Ct) and −17 °C (A). It has been described that substituting water and/or integrating organic solvents, such as glycerin, can lower the freezing point [89,90]. In this paper, we demonstrate that not only the integration of glycerin, but also the synergy between the other components, allows the freezing point to be lowered more than that of the polymer base alone (Ct).

Compared to other studies, our study achieved a functional hydrogel with a lower glycerin concentration, demonstrating a novel approach for hydrogel innovation [89,91,92].

#### 2.3.6. Adhesion to Different Surfaces

**Figure 8:**
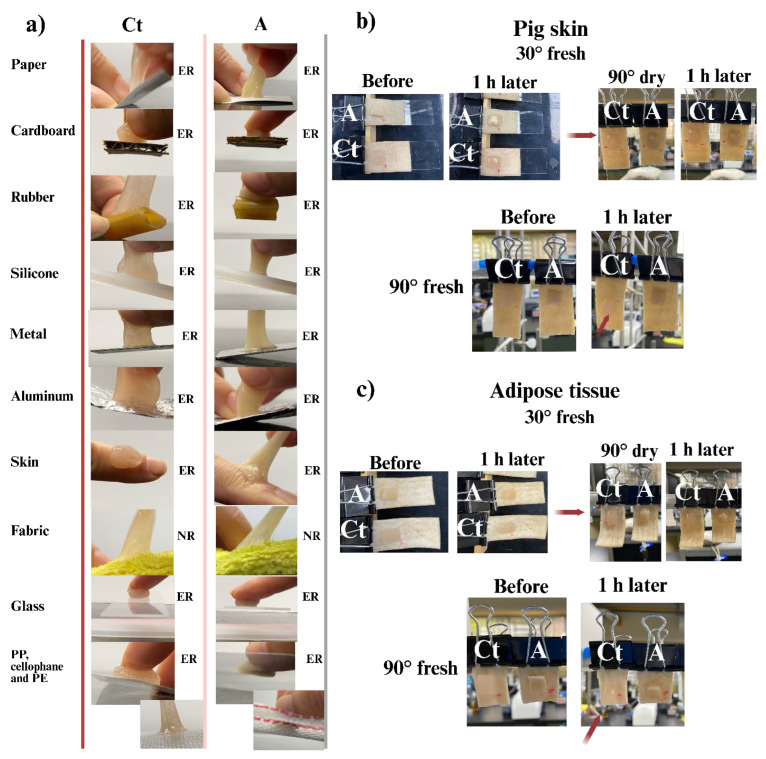
(**a**) Adhesion test on different surfaces of the evaluated hydrogels. ER is easy to remove, NR is not removable, NA does not apply; (**b**) Adhesion and gravity resistance tests to the hydrogels evaluated at 30° and 90° angles in pig skin and (**c**) Adhesion and gravity resistance tests to the hydrogels evaluated at 30° and 90° angles in pig adipose tissue. Red arrows indicate a displacement of the hydrogel.

A small sample of each hydrogel was subjected to contact with several polar and nonpolar surfaces (paper, cardboard, aluminum, silicone, fabric, rubber, metal, polypropylene, cellophane, polyethylene, glass, human skin, pig skin, and pig adipose tissue) as shown in Figure 8. Both hydrogels adhered to all the different surfaces tested. However, although they were able to attach to nonpolar substrates, this adhesion was subtle and superficial, as the hydrogels were easily removed without leaving any residue, highlighting a preference for biotic and abiotic polar substrates. In the fabric adhesion test, both Ct and A hydrogels showed firm adhesion, due to the entanglement of the fibers within the hydrogel network, which increased the effective contact area. The hydrogels stretched under mechanical stress but could not be fully removed from the fabric.

On the other hand, the remaining surfaces allowed the hydrogels to adhere and detach without leaving any residue, thereby preserving the integrity of both the hydrogels and tested substrates. The Ct hydrogel exhibited superior adhesion compared to A, due to its higher number of free functional groups capable of forming bonds through multiple mechanisms with various surfaces. In contrast, hydrogel A, in addition to possessing a certain number of functional groups designed for adhesion, plays a key role in maintaining the active ingredients and other materials in harmony within the polymer structure.

On the other hand, adhesion tests were also performed on pig skin and adipose tissue at angles of 30° and 90°, to observe the effect of gravity and to verify their adhesive and cohesive capacity. For the 90° tests, two conditions were used: samples first tested at 30° and then repositioned to 90° after one hour, and samples tested directly at 90°.

In the 30° test on pig skin (Figure 8b), the Ct hydrogel moved 2 mm, and the A hydrogel moved 3 mm. Once the test was completed, the samples were tilted to 90°, and neither of them moved. On the other hand, in the 90° test on fresh tissue, hydrogel A only moved 1 mm, while Ct moved 1 cm. Both hydrogels peeled off easily and did not reattach. In this type of tissue, hydrogel A demonstrated superior adhesion and cohesion, as it resisted deformation and stretching under the effect of gravity.

In the adhesion test on adipose tissue (Figure 8c), hydrogel A was the only sample that exhibited movement of 1 mm at 30°. At 90°, Ct moved 3 mm, which is minimal considering the test conditions, while A remained stationary. Lastly, at 90° on fresh tissue, Ct moved 5 mm and A moved 3 mm. It should be noted that hydrogel A adhered strongly to adipose tissue and was able to reattach after detachment. Adipose tissue is composed of adipocytes surrounded by a thick basal lamina containing collagen IV as the main component in its extracellular matrix, similar to bone and cartilage tissue [93]. The compatibility and composition of hydrogel A (presence of gelatin and collagen) make it highly suitable for bonding with such tissue, thus achieving stronger interactions with lipid compounds.

On the skin, the hydrogels adhered easily without causing irritation or inflammation (in this pilot study) and could be peeled off easily and completely once dry, without causing pain or damage after 6 to 10 h of contact. In general, the hydrogels could stretch without breaking, demonstrating elastic deformation resistance and consistent adhesion after repeated movement. On the other hand, when the hydrogels completely dried on the skin, they generated a protective layer that was able to stretch along with the skin, without causing pain or discomfort. Upon removal of the hydrogels, the skin appeared smoother and moisturized due to the diffusion of the active ingredients and water content in the hydrogels.

**Figure 9:**
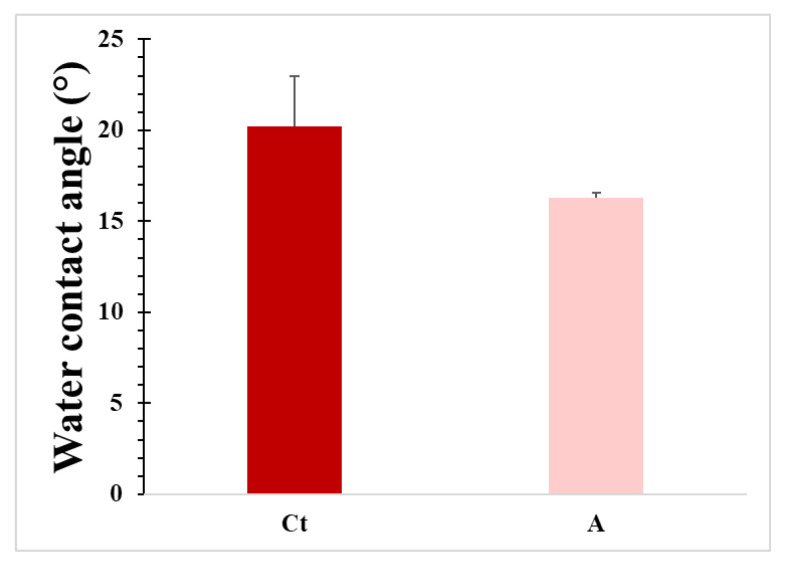
Contact angles of hydrogel Ct and hydrogel A. Data are presented as mean ± SD, *n* = 3.

On the other hand, the contact angle is a critical parameter for evaluating the degree of hydrophobicity of hydrogels, which in turn can be correlated with wettability, exudate absorption capacity in wounds, and cell adhesion [94]. Figure 9 shows the contact angles of Ct (20.24°) and A (16.31°) hydrogels. Contact angles < 90° indicate that the material surface is hydrophilic, where the values obtained are related to the amount of OH^−^ groups, among others of great hydrophilicity.

Such pronounced hydrophilicity facilitates rapid water spreading on the hydrogel surface, promoting efficient absorption of wound exudates and maintaining a moist healing environment, an essential factor for tissue regeneration. In addition, high wettability enhances the intimate contact between the hydrogel and the skin, improving adhesion and potentially contributing to a more sustained interaction or gradual diffusion of bioactive components.

### 2.4. Cell Testing

#### Hydrogel Cytotoxicity and Scratch Test

**Figure 10:**
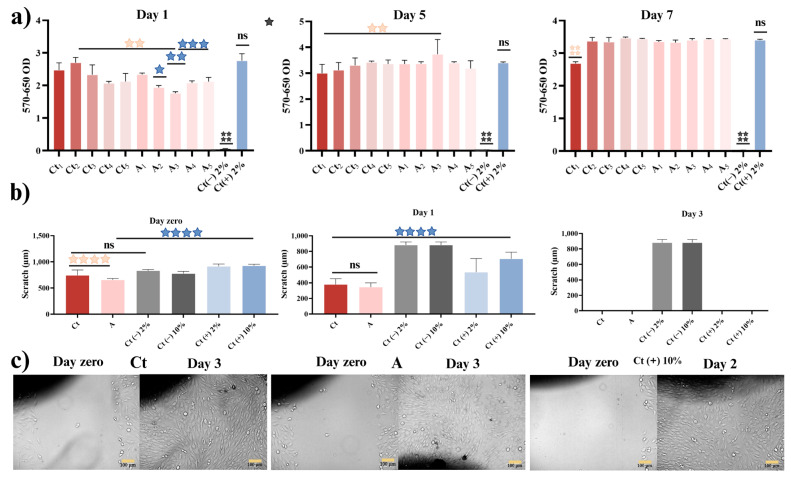
(**a**) Cytotoxicity assays on MG-63 cells in experimental hydrogels during 1, 5 and 7 days. The concentration of Ct and A hydrogel was 1 mg mL^−1^, 0.5 mg mL^−1^, 0.25 mg mL^−1^, 0.125 mg mL^−1^ and 0.0625 mg mL^−1^. (**b**) Scratch exposure after 0, 1 and 3 days of treatment with Ct, A hydrogel, control (−) and control (+) with 2% and 10% of SBF. (**c**) Representative images of MG-63 cells migration after 0, 1 and 3 days of treatment with Ct, A hydrogel, control (−) and control (+), scale bar = 100 μm The scale bar is 100 µm. (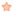) Show a significant difference compared between A and Control (Ct) hydrogels, (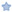) compared between Ct and A with Ct (+), (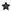) compared between Ct (−) and Ct (+). No significant difference (ns). Data are presented as mean ± SD, *n* = 4. Statistical analysis was carried out using a one-way ANOVA followed by a post hoc Tukey test (* *p* < 0.05, ** *p* < 0.01, *** *p* < 0.001, **** *p* < 0.0001).

To evaluate the cytotoxic behavior of the experimental hydrogels, human osteoblasts (MG-63 cells) were tested using concentrations of 1, 0.5, 0.25, 0.0125, and 0.0065 mg mL^−1^ of the Ct and A hydrogels. The results, as shown in Figure 10a, indicated that the material in Ct presented a higher cellular activity compared to hydrogel A, but without a significant difference to Ct (+) during day 1. On day 5, hydrogel A with a concentration of 0.25 mg mL^−1^ (A_3_) achieved a better metabolic response than hydrogel Ct, but there was no significant difference with Ct (+). Lastly, on day 7 of the test, all the concentrations on both hydrogels, except Ct 1 mg∙mL^−1^ (Ct_1_), had similar values to Ct (+), suggesting that the hydrogels are not cytotoxic and that their viability increases as the application time passes.

The comparative MTT assays of the individual components and the complete formulation (Figure S7) revealed clear differences in cellular metabolic activity. All tested samples maintained high osteoblastic viability, confirming the absence of cytotoxic effects. However, Hydrogel A exhibited the highest and most consistent metabolic activity across all evaluated concentrations, comparable to or exceeding the control group. In contrast, the base hydrogel (Ct) maintained good viability but showed a slight reduction at lower concentrations, indicating that the active components contribute significantly to cellular stimulation. The Mn-HA system presented a pronounced metabolic peak at 50%, while ZnP maintained stable but moderate viability, suggesting that both are biocompatible but act through different mechanisms. The vitamin group exhibited increased metabolic activity at higher concentrations (50%), supporting their known role in osteoblast metabolism. Altogether, these findings indicate that the incorporation of Mn-HA, ZnP, and vitamins within the polymeric matrix results in a synergistic enhancement of cellular metabolism and proliferation, evidencing the cooperative effect of these bioactive components in promoting osteoblastic activity. This behavior aligns with previous studies reporting complementary biological effects of these ions in osteogenic environments [19,95].

According to the scratch test (Figure 10b), the hydrogels exhibited significant differences between each of the experimental groups. However, at the end of the first day of testing, the hydrogels did not show significant differences between them (wound closure of 80.73% in Ct and 73.81% in A), but there were noticeable differences with the positive controls, indicating a greater proliferation and, thus, greater wound closure in the monolayer. On day two, the positive controls achieved complete closure of the wound, while the evaluated hydrogels achieved it on day three. On day two, the hydrogels achieved wound closure rates of 97.39% and 94.52% for Ct and A, respectively. It is important to emphasize that the environment induced by hydrogels plays an important role in cell adaptation for osteoblast proliferation. Moreover, the first 24 h are critical for cell adhesion processes; they are also important for cells to react to the experimental medium [96]. We consider that this adaptation process corresponds to the first and second day of the test, where the cells respond better to the stimulus caused by the hydrogels, as it corresponds to the MTT assays mentioned above.

Comparing the morphology between the cells in each treatment (Figure 10c), hydrogel Ct showed greater proliferation and interactions between cells with similar morphology to that of the control (+). For their part, the cells in the sample of hydrogel A presented a morphology that is characteristic of osteoblasts undergoing cell differentiation, with a defined nuclear envelope, few vacuole formations, and elongated, fusiform morphology. This resulting characteristic suggests that the components present in the hydrogel, such as collagen, chondroitin sulfate, ZnP, and the Mn-Ha itself, function as cofactors for regeneration and bone differentiation [17,34,97,98,99,100,101]. In fact, the vitamins used by themselves can stimulate the osteoblasts, increase calcium absorption, stimulate and sustain cartilage, maintain normal bone density levels, and decrease osteocalcin by directly intervening with osteoclasts [13,102,103].

These results show that both the hydrogel base (Ct) and the hydrogel with the active ingredients (A), which include collagen, chondroitin sulfate, ZnP, and Mn-Ha, are beneficial for the cells studied. These compounds allow the cells to adapt, differentiate and be able to proliferate and regenerate wounds [10,104,105,106,107,108]. Future work will include assays such as alkaline phosphatase (ALP) activity, Runx2/OCN/OPN expression, and Alizarin Red staining to corroborate and support the osteogenic functionality of the hydrogel.

## 3. Conclusions

A hydrogel integrating a biopolymer network with vitamins, nanoparticles, and other active ingredients was developed. It exhibited favorable physicochemical characteristics, including swelling capacity, efficient water absorption, controlled biodegradability, and responsiveness to external stimuli. Moreover, the material demonstrated self-healing ability and good adhesion to different surfaces, such as skin and adipose tissue. The hydrogel also showed excellent cytocompatibility, supporting cell viability and suggesting potential to promote cell regeneration. The synergistic combination of its components appears to favor osteoblastic functionality under in vitro conditions. However, further biological validation remains necessary, particularly assays related to osteogenic differentiation, such as ALP activity, mineralization, and osteogenic gene expression, to confirm and elucidate its osteogenic potential.

Overall, the results indicate that this intelligent and multifunctional hydrogel represents a promising platform for future applications in tissue rehabilitation and regenerative medicine, particularly in bone-related therapies, pending comprehensive in vitro and in vivo evaluation.

## 4. Materials and Methods

Materials: The manganese chloride tetrahydrate (MnCl_2_∙4H_2_O), zinc chloride (ZnCl_2_), monopotassium phosphate (KH_2_PO_4_), 96% ethanol (EtOH) and polyvinyl alcohol (PVA) were from FAGALab, Mexicali, Baja California, Mexico. Hydrochloric acid (HCl) and ammonium hydroxide (NH_4_OH) from J.T. Baker, Randor, PA, USA, chitosan (CS) from Sigma Aldrich, Carlsbad, CA, USA, guar gum (GG), vegetable glycerin (Gli), sodium borate (Bx), hydrolyzed collagen (Col), hammamelis hydrolate (Hamm), calendula glycolic extract (Cal), BASF HYALUROSMOOTH^®^ Active (HA), D-panthenol (Dp), BASF VITA A LIKE^®^ Active (ABA) and soy lecithin (Lec) from Cosmopolitan, glucosamine HCl (Glu), chondroitin sulfate (Con), vitamin A (VitA), vitamin E (VitE), vitamin K2 (VitK) and vitamin D3 (VitD) from BulkSupplements, Henderson, NV, USA, olivoil avenate (Ol) and Kemnat^®^ from Cosmat, Azcapotzalco, Distrito Federal, Mexico, tannic acid (TA) from Jalmek, San Nicolás de los Garza, Nuevo León, Mexico; sodium hydroxide (NaOH) from Fermont, Mexico; Cocobetaine (CC) from Chemie NRW, Mexicali, Baja California, Mexico; gelatin (Gel) from Duche, Mexico, and distilled water from Hwater, Mexicali, Baja California, Mexico. A solution of sterile PBS (Phosphate-buffered saline) 1× pH 7.2 and a solution of SBF (Simulated Body Fluid) pH 7.4 was prepared. The zinc phosphate (ZnP) micro/nano particle synthesis, as well as for manganese-doped hydroxyapatite (Mn-Ha) synthesis and characterization, are shown in the Supplementary Materials.

**Figure 11:**
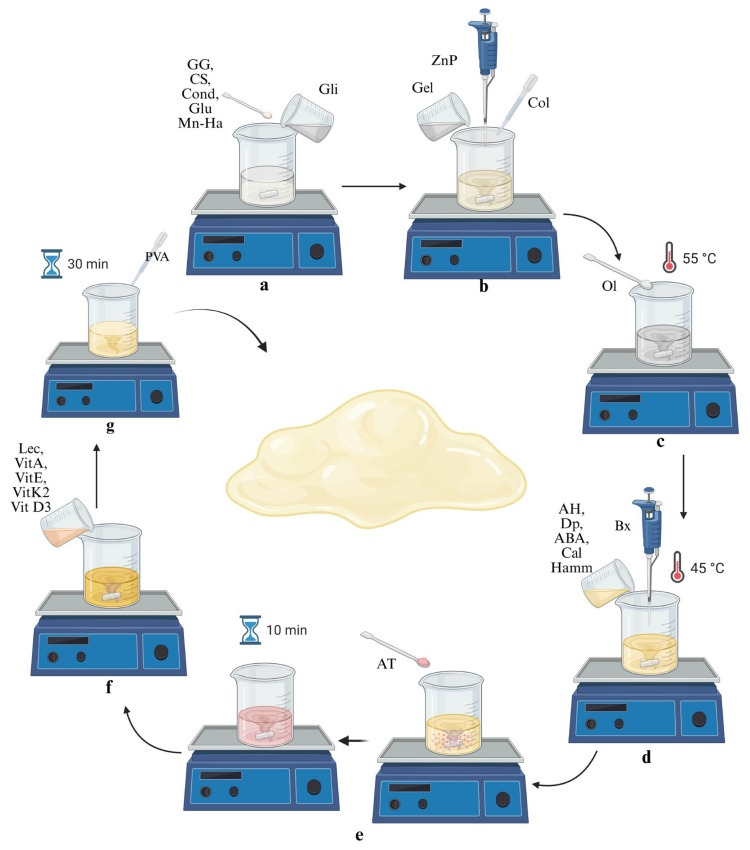
Schematic representation of the Hydrogel synthesis process: The reagents were sequentially added and mixed. Initially, GG, CS, Cond, Glu, Mn-Ha, and Gli were homogenized at room temperature (pH 5.6) (**a**), followed by the addition of Gel, Col and ZnP (pH 6.6) (**b**). The mixture was then heated to 55 °C, and reagent Ol was incorporated (**c**). After cooling to 45 °C, reagents AH, Dp, ABA, Cal, Hamm, Bx, and AT were successively added and homogenized (pH 6.5) (**d**,**e**). Subsequently, reagents Lec, VitA, VitE, VitK2, and VitD3, pre-mixed in a separate container, were combined with the main solution (**f**), and finally PVA solution was added under stirring for 30 min to obtain the final hydrogel (pH 6.5) (**g**).

Hydrogel Synthesis: The order of addition, mixing and quantities of the reagents is followed according to Table S1. First, the reagents from 1 to 6 were mixed at 200 rpm for 5 min to get rid of any lumps at room temperature (pH 5.6) (Figure 11a). Then, the necessary amount of solutions of reagents 7 to 9 was added, allowing each to mix before adding the next one (pH 6.60) (Figure 11b). Then, the mixture was heated at 55 °C, and then reagent 10 was added. The mixture was left stirring at 300 rpm until the emulsifier was fully incorporated (Figure 11c). The heat source was removed, and the mixture was cooled to 45 °C. Then, reagents 11 to 18 were sequentially added, allowing their homogenization in the mixture (pH 6.51) (Figure 11d). The reagent 18 was slowly added under stirring for 10 min in order to avoid the creation of lumps and obtain a homogeneous solution (pH 6.24) (Figure 11e). Reagents 19 to 23 were mixed by hand in a separate container and later added to the main solution (pH 6.20) (Figure 11f). Finally, reagent 24 was added and allowed to mix with the hydrogel for 30 min under the same stirring rate and temperature (final pH 6.50) (Figure 11g).

### 4.1. Hydrogel Structure Characterization

Scanning Electron Microscopy (SEM) Energy Dispersive X-Ray Spectroscopy (EDS): The hydrogels were characterized by SEM-EDS (JeolJSM6010LA, JEOL Ltd., Akishima, Tokyo, Japan), by preparing 1 cm^2^ samples previously dehydrated in absolute alcohol for 24 h. Then, the samples were left in a desiccator for 24 h to achieve complete dryness. Once they were dry, the samples were mounted on a double-sided carbon adhesive tape. The samples were analyzed under low vacuum (30 Pa) at 15kV, a working distance of 10 mm, and a spot size of 50 using a backscattered electron detector.

X-ray fluorescence (XRF): The XRF analysis was carried out using a Shimadzu spectrometer (Shimadzu-EDX 7000, Shimadzu Corporation, Kyoto, Japan) equipped with a Rh X-ray tube operating at 30 kV. Prior to measurement, approximately 1.5 g of hydrogel A was placed in a 60 mm sample holder for subsequent measurements.

FTIR: Fourier transform infrared spectroscopy (FTIR, PerkinElmer Frontier, PerkinElmer, inc. Waltham, MA, USA) was used to characterize the hydrogels’ functional groups. Freshly prepared hydrogels were stored at −20 °C for 24 h, then placed in a desiccator for 5 days before analysis. Each spectrum was measured over a wavenumber range of 4000 to 400 cm^−1^ with a resolution scan of 1 cm^−1^ [109].

AFM: The three-dimensional structure, roughness and contrast of the hydrogels were analyzed at room temperature by Atomic Force Microscopy (AFM) (Park Systems Corporation, NX10, Suwon, Republic of Korea) equipped with an acoustic enclosure and a vibration isolation table (Thorlabs, PFR90150, Newton, NJ, USA). The substrates were cut to 1 × 1 cm^−2^ and mounted on glass slides (previously washed with soap and water, rinsed with abundant distilled water and finally with 98% ethanol). The slides were covered and kept in a desiccator until use. Then, 1 drop of hydrogel supplied by a 21 G needle syringe was deposited on each slide. The slides were placed in a centrifuge at 5100 rpm for 3 min and finally left in a desiccator until further analysis. The tests were conducted using a PPP-NCHR tip (AC160TS, APM company, Seoul, Republic of Korea) with a constant force of 42 N m^−1^, resonance frequency of 330 kHz, and a scan rate of 0.60 Hz in non-contact mode. The scanning area was 25 µm^2^. XEI 5.2.0 software (Park Systems, Republic of Korea) was used for data analysis, and the arithmetic mean roughness parameter (Ra) was analyzed.

Moisture content: The humidity content was obtained from 2 cm^2^ samples of each hydrogel, where their initial weight was taken. The samples were then kept for 5 days in a desiccator before a second weight reading. The humidity content was calculated using the following equation [50]:

(4) $$Humidity\;content\;\left(\%\right)=\left(\frac{W_{1}-W_{2}}{W_{1}}\right)\times 100$$

where *W*_1_ is the initial weight and *W*_2_ is the weight of the dried sample. Measurements were performed in triplicate for each hydrogel.

Absorbed moisture content: The hydrogels were sectioned into 2 cm^2^ pieces, and their initial moisture content was determined. The samples were then immersed in a container with 4 mL of distilled water for 3 h to prevent dissolution of the gel. Following this period, the samples were placed in a desiccator to eliminate surface moisture, and their final weight was recorded. The absorbed moisture content was calculated using the following equation:

(5) $$Absorbed\;humidity\;content\;\left(\%\right)=\left(\frac{W_{2}-W_{1}}{W_{1}}\right)\times 100$$

where *W*_1_ is the initial weight, and *W*_2_ is the final weight. Measurements were performed in triplicate for each hydrogel. The test was repeated using the dried hydrogels.

Swelling test: The swelling behavior of the hydrogels was measured using the methodology described by Perez-Diaz et al. [110]. Initially, the pre-weighed hydrogels were immersed in 4 mL of PBS at room temperature. The absorbed PBS was quantified at 30 min intervals during 2 h with an analytical balance until equilibrium was achieved. Between measurements, the PBS solution was removed and replaced with a fresh one. This procedure was repeated at extended intervals of 4, 8, and 24 h. The swelling ratio of the hydrogels was calculated using the following method:

(6) $$Swelling\;degree\left(\%\right)=\frac{W_{2}-W_{1}}{W_{1}}\times 100$$

where *W*_2_ is the wet hydrogel weight and *W*_1_ is the dried hydrogel weight. Measurements for each hydrogel were made in triplicate.

Rheological Studies of Hydrogels: Rheological measurements were carried out using a rheometer (Anton Parr MCR102, Anton Paar GmbH, Graz, Austria) at room temperature. Hydrogel disks of 25 mm diameter and 2.5 mm height were prepared for frequency sweep, strain sweep and viscosity measurements. The frequency sweep (ω) was conducted between 1 and 100 rad s^−1^, which measured the variation in storage modulus (G′) and loss modulus (G″). The amplitude sweep measures the strain dependence (1–1000%) in G′ and G″, allowing for the calculation of the stress dependence and yield strength of hydrogels.

### 4.2. Hydrogel Characterization—External Stimuli

Solubility: To measure the solubility of the evaluated hydrogels (g hydrogel/100 g^−1^ solvent), 1 cm^2^ samples were cut and weighed (*W*_0_). Subsequently, the hydrogels were placed within 10 mL of distilled water, PBS, 70% *w*/*v* EtOH, 1 N HCl, 1 N NaOH and SBF separately for 1 day; the presence of a solid-gel material within the containers was observed. The liquid was filtered using a Whatman #4 filter under vacuum, and the remaining solid was weighed after leaving it to dry for 1 day in a desiccator (*W*_1_).

(7) $$solubility\;\left(\frac{g}{100g}\right)=\left(W_{0}-W_{1}\right)\times \left(\frac{100}{Vol_{solvent}\times \rho_{solvent}}\right)$$

Hydrogel Biodegradability (in vitro): To evaluate the biodegradability of the fabricated hydrogels, the weight loss test was used in different periods of time, up to a maximum of 28 days, with intervals of 7 days (1, 7, 14, 21 and 28 days). For this purpose, the prepared 1 cm^2^ hydrogels were immersed in PBS phosphate buffer (10 mL) and kept at 37 °C. Every three days, the medium was changed to a new one and the test tube was slightly shaken. The following equation was used to calculate the weight loss percentage [111,112].

(8) $$Biodegradability\;\left(\%\right)=\frac{W_{1}-W_{2}}{W_{1}}\times 100$$

In the previous equation, *W*_1_ represents the initial sample weight, and *W*_2_ represents the dry weight after being extracted from PBS. The average value of three samples for each hydrogel is reported.

Bioactive Study—Behavior in normal and abnormal SBF: To evaluate the hydrogel’s behavior when conditions resemble those present in wounds or injuries, the previously described weight loss test is used for 21 days, with 7 day intervals (1, 7, 14 and 21 days) while also inspecting changes in the medium’s pH, using 10 mL of SBF medium at normal pH (7.4) and in slightly acidic conditions (pH 6.7) at 37 °C [73,74,112].

Adhesion to different surfaces: Following the method of X. Su et al. [113], with slight modifications, small hydrogel samples were tested for adhesion on various surfaces (e.g., paper, cardboard, aluminum, silicone, fabric, rubber, polypropylene, cellophane, polyethylene, glass, human skin, pig skin, and adipose tissue) under standard temperature and pressure. The hydrogels were then applied to the skin for 10 h to observe their effects and any changes in the hydrogels. The pig skin and adipose tissue samples were obtained from a local meat market; therefore, ethical approval was not required.

In addition, samples of the hydrogels were placed in 1 cm^2^ pieces on pig skin and pig adipose tissue at a 30° inclination for 1 h. After the time had passed, the tissues with the adhered hydrogels were placed at 90°. Finally, the test was repeated by placing the tissues at 90°, and the fresh hydrogels were contacted to check their adhesive and cohesive capacity and the effect of gravity on them.

Water adhesion: A small hydrogel sample was introduced into 20 mL of water at room temperature without any additional treatment [113]. Subsequently, the hydrogel was pressed with a fingertip (pressure ~ 1 kPa) for 10 s, and then the external pressure was removed immediately to observe its adherence capacity. The experiment was repeated, but the hydrogel was pressed outside of water. Once adhered, it was introduced into water, and it was observed if the hydrogel detached.

Self-healing and self-recovery abilities of hydrogel: The hydrogel’s self-repair ability was tested by cutting it into two parts, one with Green PGR7-L dye (Cosmopolitan, Ciudad de Mexico, Mexico). The pieces were rejoined and retained their shape and checked with opposing movements to ensure they held together [114,115]. Additionally, self-healing was tested by cutting the surface of the hydrogels and observing the time taken to return to its original shape without external stimuli [39]. The recovery and self-healing tests were conducted using a stereoscope (Leica, DMIL, Leica Microsystems, Wetzlar, Germany) with 40× magnification, and the rate of self-healing was measured by ImageJ (version 1.54g, National Institute of Health, Bethesda, MD, USA) software.

Hydrogel behavior according to temperature: Using 2 mL of each hydrogel, they were gradually heated in a water bath (1 °C min^−1^). After reaching 30 °C, the hydrogels were slightly stirred and inverted to check if they flowed, repeating for each degree increase. When flow was observed, measurements were concluded. Similarly, hydrogels were cooled to −20 °C, frozen, and then monitored while piercing with a stick. The temperature at which the hydrogels could be pierced was noted as the maximum before freezing.

### 4.3. Biological Test

Cell culture: The human osteoblast-like cell line MG-63 (ATCC, Manassas, WV, USA) was cultured using Dulbecco’s Modified Eagle medium (DMEM, Gibco, Invitrogen, Waltham, MA, USA) supplemented with 10% heat-inactivated fetal bovine serum (FBS; Gibco, Invitrogen) and 1% penicillin-streptomycin (100 Um L^−1^/100 µg mL^−1^, Gibco, Invitrogen, Waltham, MA, USA). Cells were incubated at 37 °C in a humid atmosphere containing 5% CO_2_ until they reached approximately 80% confluence [109].

MTT Assay for Cell Viability: Cell viability under treatment with experimental hydrogels was determined via the MTT colorimetric assay. MG-63 cells (ATCC, Manassas, WV, USA) were seeded at a density of 1.5 × 10^4^ cells per well in 96-well plates (Corning, New York, NY, USA) and exposed to 100 μL of hydrogel suspensions at concentrations of 0.5, 0.25, 0.125, and 0.065 mg∙mL^−1^ in triplicate for 1, 5, and 7 days. Following each incubation period, the culture medium was removed carefully, and the cells were washed gently with pre-warmed PBS. Then, 200 µL of MTT solution (5 mg mL^−1^, Sigma Aldrich, USA) was added and incubated for 3 h at 37 °C in 5% CO_2_ [116]. The MTT solution was discarded afterward, and the formazan crystals formed were dissolved in 200 µL of dimethyl sulfoxide (Sigma Aldrich, Carlsbad, CA, USA) while shaking at 200 rpm, in the dark, for 20 min. Absorbance was measured at 590 nm using a microplate reader (Thermoskan, Thermo Fisher Scientific, Carlsbad, CA, USA). Wells containing only culture medium were used as blanks, and untreated cells served as negative controls for cytotoxicity [116].

Scratch Test: Migration capability was evaluated by seeding MG-63 cells at 2.5 × 10^4^ cells per well in 12-well plates (Corning, New York, NY, USA) and culturing until full confluence was achieved. A linear scratch was created by scraping the monolayer with a 200 µL pipette tip [117]. Detached cells were removed by washing three times with warm PBS, then fresh DMEM containing 5% FBS and hydrogel at 1 mg mL^−1^ was applied. Control groups used 2% and 10% FBS. Wound closure was documented at 0, 24 h, 48 h and 72 h using a digital phase-contrast microscope (ZOE, Bio-Rad, Irvine, CA, USA). The migration rate was quantified by calculating the decrease in wound width over time using ImageJ software (version 1.54g, National Institute of Health, Bethesda, MD, USA).

Statistical Analysis: All quantitative data are expressed as mean ± standard deviation (SD) from at least three independent experiments performed in triplicate. For both the MTT and Scratch assays, a minimum of four independent studies per group were conducted. Statistical significance among groups was evaluated using were carried out using one-way ANOVA followed by Tukey’s post hoc test via GraphPad Prism 9 (GraphPad Software Inc., San Diego, CA, USA). A *p* < 0.05 was considered statistically significant [109].

## Data Availability

The data presented in this study are available upon request from the corresponding author due to ongoing related research and confidentiality agreements.

## Supplementary Materials

The following supporting information can be downloaded at: https://www.mdpi.com/article/10.3390/gels11110910/s1, Figure S1: Representative diagram of Mn-Ha synthesis. Figure S2: (a) Pore size distribution in hydrogel A by SEM; (b) SEM micrograph of ZnP and its size distribution; and (c) EDX analysis composition of ZnP. Figure S3: XRF analysis composition of hydrogel A. Figure S4: (a) Micrograph and elemental results of Mn-Ha by SEM-EDS, (b) Size distribution of both Ha and Mn-Ha. Figure S5: Size distribution of the protuberance in hydrogel A according to AFM. Figure S6: (a) Representative flow curve (Shear stress vs. Shear rate), (b) complex viscosity vs. shear rate of hydrogel Ct and A. Figure S7: Cytotoxicity assays on MG-63 cells in experimental hydrogels for 1 day. The concentration of Ct, A, Ct + Mn-Ha, Ct + ZnP, and Ct + vitamins hydrogels were 100%, 50%, 25%, 12.5%, 6.25%. () Show a significant difference, *p* < 0.05, using one-way ANOVA followed by a post hoc Tukey test. No significant difference (ns). (* *p* < 0.05, ** *p* < 0.01, *** *p* < 0.001, **** *p* < 0.0001). The scale bar is 100 µm. The error bars represent SD, *n* = 4. Table S1: Composition of hydrogels Control and A. References [118,119,120,121] are cited in the Supplementary Materials.
