# Supplementary material for: Synergistic and Intelligent Hydrogel for Conducting Osteoblast Proliferation: Synthesis, Characterization, and Multifunctional Properties
Source: Gels. 2025 Nov 14;11(11):910. doi: 10.3390/gels11110910 (PMC12652084; doi:10.3390/gels11110910)
Supplement: Supplementary file 1 [file gels-11-00910-s001.zip › gels-3976640-supplementary.pdf]

Supplementary material

"Synergistic and intelligent hydrogel for conducting osteoblast proliferation: Synthesis, characterization, and multifunctional properties"

Karen Michelle Guillén-Carvajal*†, Benjamín Valdez-Salas*†, Ernesto Alonso Beltrán-Partida†, Jorge Salomón Salvador-Carlos, Mario Alberto Curiel-Álvarez, Jhonathan Castillo-Saenz, Daniel González-Mendoza, and Nelson Cheng

K. M. Guillén-Carvajal, B. Valdez-Salas, E. A. Beltrán-Partida, J. S. Salvador-Carlos, M. A. Curiel-Álvarez, and J. Castillo-Saenz

Core Facility- Química y materiales avanzados, Instituto de Ingeniería, Universidad Autónoma de Baja California, Blvd. Benito Juárez and Normal s/n., 21280 Mexicali, Baja California, México

D. González-Mendoza

Instituto de Ciencias Agrícolas, Universidad Autónoma de Baja California, Carretera a Delta s/n C.P. 21705, Ejido Nuevo León, Mexicali 21100, BC, México

N. Cheng

Magna International Pte Ltd., 10 H Enterprise Road, Singapore 629834, Singapore

K. M. Guillén-Carvajal

E-mail: kguillen@uabc.edu.mx

ORCID: https://orcid.org/0000-0002-1180-6523

Benjamín Valdez-Salas

E-mail: benval@uabc.edu.mx

ORCID: https://orcid.org/0000-0002-6788-7545

1. Experimental Section

Materials: The manganese chloride tetrahydrate ($MnCl_2 \cdot 4H_2O$), zinc chloride ($ZnCl_2$), monopotassium phosphate (KH_2PO_4), and polyvinyl alcohol (PVA) were purshased from FAGALab, Mexico; hydrochloric acid (HCl), ammonium hydroxide (NH_4OH) and nitric acid (HNO_3) from Baker, Mexico; chitosan (CS) from Sigma Aldrich, US; guar gum (GG), vegetable glycerin (Gli), sodium borate (Bx), hydrolyzed collagen (Col), witch hazel hydrolate (Hamm), calendula glycolic extract (Cal), BASF HYALUROSMOOTH® active (HA), D-panthenol (Dp), BASF VITA A LIKE® Active (ABA) and soy lecithin (Lec) from Cosmopolitan; glucosamine HCl (Glu), chondroitin sulfate (Con), vitamin A (VitA), vitamin E (VitE), vitamin K2 (VitK) and vitamin D3 (VitD) from BulkSupplements, US; olivoyl avenate (Ol) and Kemnat® from Cosmat, Mexico; tannic acid (TA) from Jalmek, Mexico; sodium hydroxide (NaOH), calcium hydroxide ($Ca(OH)_2$) and diammonium phosphate ($(NH_4)_2HPO_4$) from Fermont, Mexico; Cocobetaine (CC) from Chemie NRW, Mexico; gelatin (Gel) from Duche, Mexico; and distilled water from Hwater, Mexico.

Chemical synthesis of zinc phosphate micro/nanoparticles (ZnP): The zinc phosphate micro and nanoparticles (ZnP) were synthesized and characterized according to our previous work [1]. Briefly, 2.5 mL of the 1% Cocobetaine solution was taken and left in an ultrasonic bath for 5 min at a frequency of 22 kHz and 40% amplitude. Then, 5 mL from a 0.05 M $ZnCl_2$ solution in aqueous medium and pH 4 regulated with 1 M HCl was added to this solution. It was left to mix for 10 min, and then 5 mL of a 0.025 M KH_2PO_4 solution was added dropwise and left to react for 10 min. The final pH was adjusted to pH 8.5 with 10% ammonium hydroxide and left to react for another 10 min. Lastly, it was ultrasonicated for 10 min using an ultrasonic homogenizer (OMNI SONIC RUPTOR 400) at 22 kHz of frequency and 40% amplitude. The precipitate was collected and washed three times with water/ethanol using a centrifuge at 6,000 rpm for 10 min. After the last wash, they were left to dry in a desiccator for 48 h at room temperature and then resuspended.

Synthesis of manganese-doped hydroxyapatite (Mn-Ha): Following the method proposed by Villaseñor et al., [2] manganese-doped hydroxyapatite is synthesized using a hydrothermal method. A 2%w/v $MnCl_2 \cdot 4H_2O$ solution was prepared by diluting 0.2 g in 10 mL of distilled water. Consequently, 8 g of $Ca(OH)_2$ were dissolved in 30 mL of distilled water. The $MnCl_2$ was added dropwise to the $Ca(OH)_2$ solution, with constant stirring, and similarly, a solution of $(NH_4)_2HPO_4$

(12.33 g in 30 mL) was added gradually (dropwise) to the $Ca(OH)_2/MnCl_2$ solution. Subsequently, 50 mL of 2M HNO_3 was added to modify the pH of the solution to 7. Next, the solution was placed in an autoclave at a temperature of 200°C for 24 h. Then, it was washed with distilled water and filtered under vacuum. Lastly, the sample was dried at 80°C for 8 h and calcined at 500°C for 3 h, producing a whitish powder with pink to violet hues. A diagram of the procedure is shown in **Figure S1**.

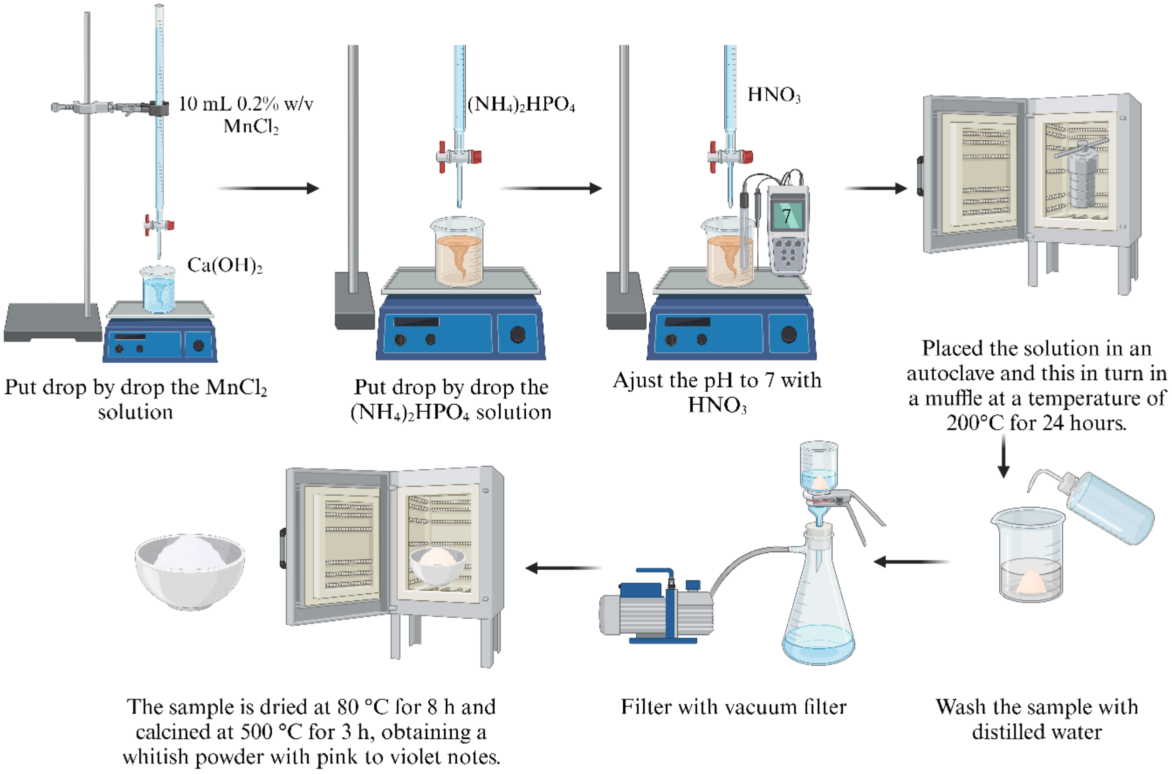

Figure S1: Representative diagram of Mn-Ha synthesis.

Characterization of ZnP y Mn-HA: The morphology, homogeneity, and size-distribution of the ZnP and Mn-HA were evaluated using Field Emission Scanning Electron Microscopy (FE-SEM; LYRA 3, Tescan, Brno, Czech Republic) at an acceleration voltage of 10 kV with a secondary electron detector. ZnP and Mn-HA were taken separately and suspended in absolute ethanol; they were sampled on double-sided adhesive carbon conductive tape, dried at room temperature, and analyzed using FE-SEM. The chemical analysis was performed by energy dispersive X-ray spectroscopy (EDX, Tescan) coupled to FE-SEM, at 10 kV with a large spot size to adjust a count

rate per second suitable for spectrum collection [3]. The diameter distribution of microparticles and nanoparticles was obtained using ImageJ 1.54 software (National Institutes of Health, USA).

Results

According to the micrographs obtained by SEM (**Figure S2b**), the ZnP had an oval flake shape with a length of ~1.76 µm and a width of ~0.20 µm. On the other hand, EDX analysis (**Figure S2c**) demonstrated the presence of Zn (57.45 wt.%), P (6.55% wt.%), O (26.32% wt.%), and traces of C (14.58% wt.%) and Cl (0.57% wt.%).

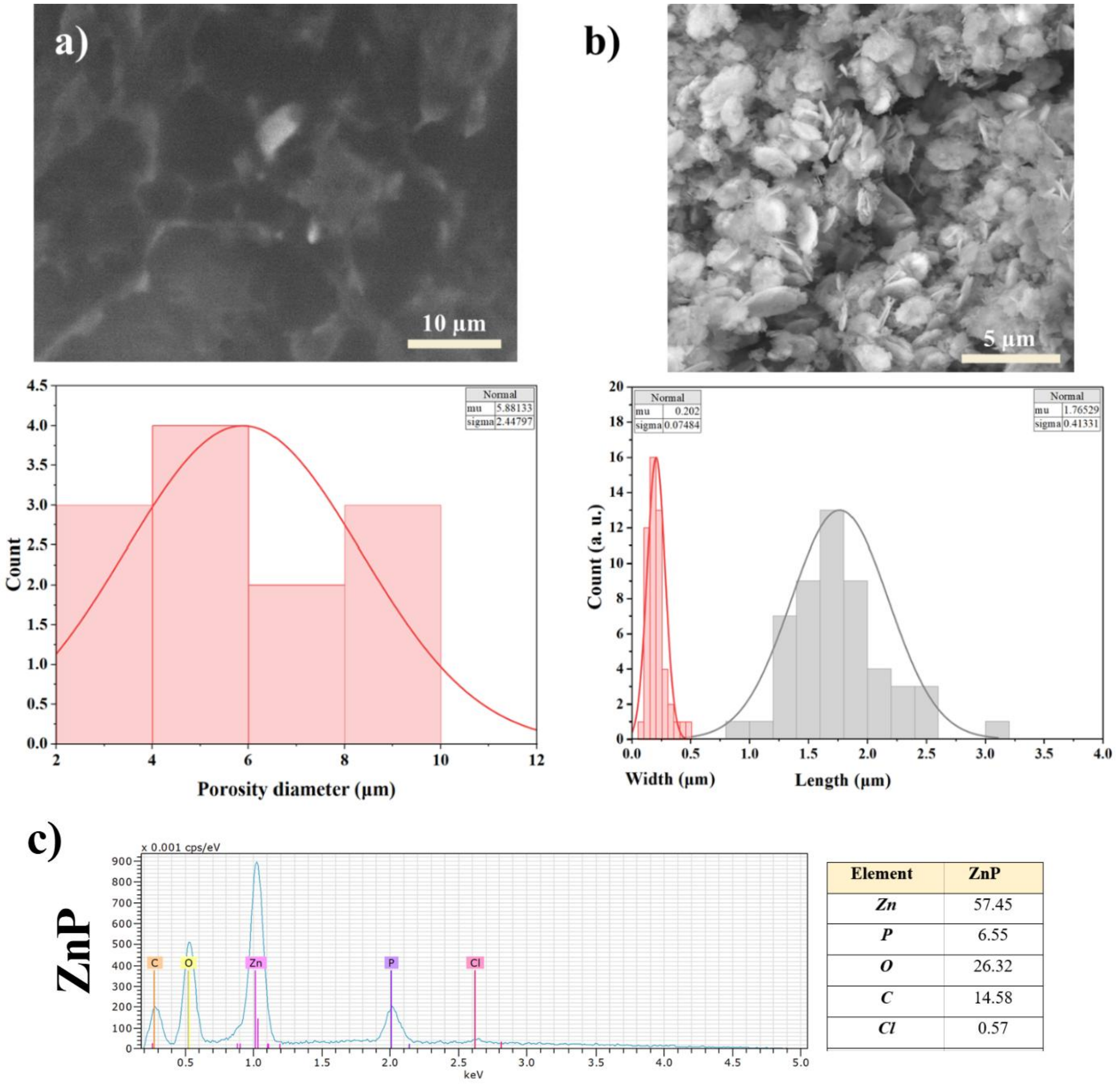

Element	ZnP
Zn	57.45
P	6.55
O	26.32
C	14.58
Cl	0.57

Figure S2: a) Pore size distribution in hydrogel A by SEM; b) SEM micrograph of ZnP and its size distribution, and c) EDX analysis composition of ZnP.

The XRF analysis shown in **Figure S3** confirms the presence of P, Ca, Mn, Zn, S, and K in hydrogel A. According to the compositional data, the (Ca + Mn)/P ratio ranged from 1.42 to 1.46, which is slightly below the typical bioabsorption threshold [2].

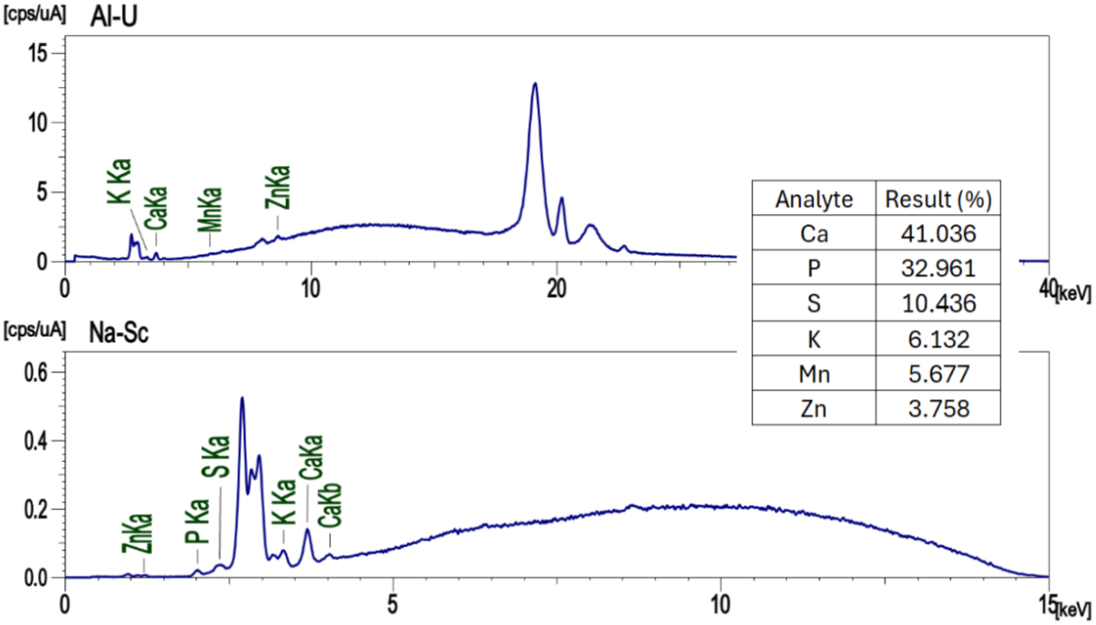

Analyte	Result (%)
Ca	41.036
P	32.961
S	10.436
K	6.132
Mn	5.677
Zn	3.758

Figure S3: XRF analysis composition of hydrogel A.

The Mn-HA were also characterized by SEM-EDX (**Figure S4a**), showing spherical structures of both hydroxyapatite and Mn-Ha. The larger structures (~254 nm) correspond to Ha, while the smaller ones (~90 nm) belong to Mn-Ha (**Figure S4b).** The stoichiometric ratio obtained for Mn-HA was suitable for bioabsorption, with a calculated (Ca + Mn)/P ratio of 1.67, according to the composition determined by EDX (Ca = 23.05 wt%, Mn = 0.44 wt%, P = 14.05 wt%) [2], [4]. In some Mn-HA samples, the composition reached Ca = 29.39 wt%, Mn = 2.11 wt%, and P = 16 wt%, resulting in a (Ca + Mn)/P ratio of 1.96. Although the Ca/P ratio was higher than the ideal stoichiometric value, these Mn-Ha can still be considered bioabsorbable, since bioceramics with Ca/P ratios close to 2.0 are generally regarded as poorly soluble but bioresorbable materials [2].

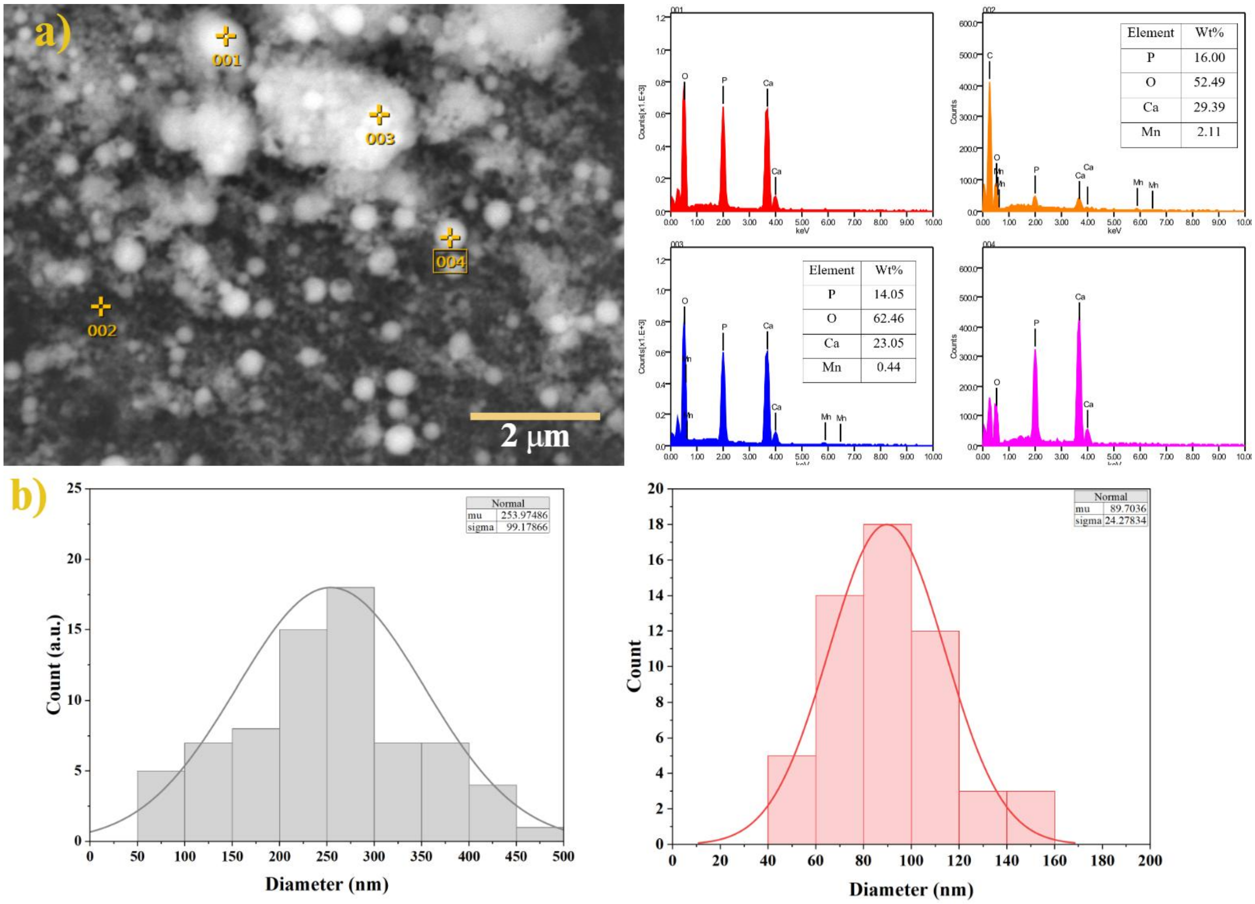

Figure S4: a) Micrograph and elemental results of Mn-Ha by SEM-EDS, b) Size distribution of both Ha and Mn-Ha.

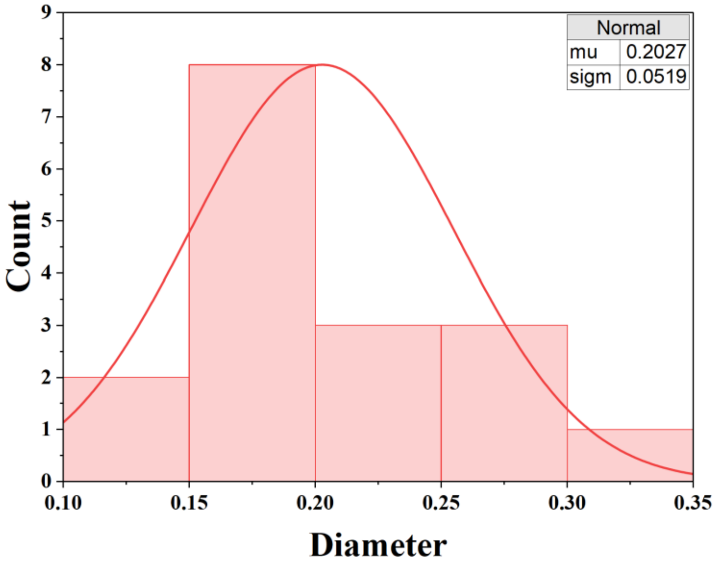

Figure S5: Size distribution of the protuberance in hydrogel A according to AFM.

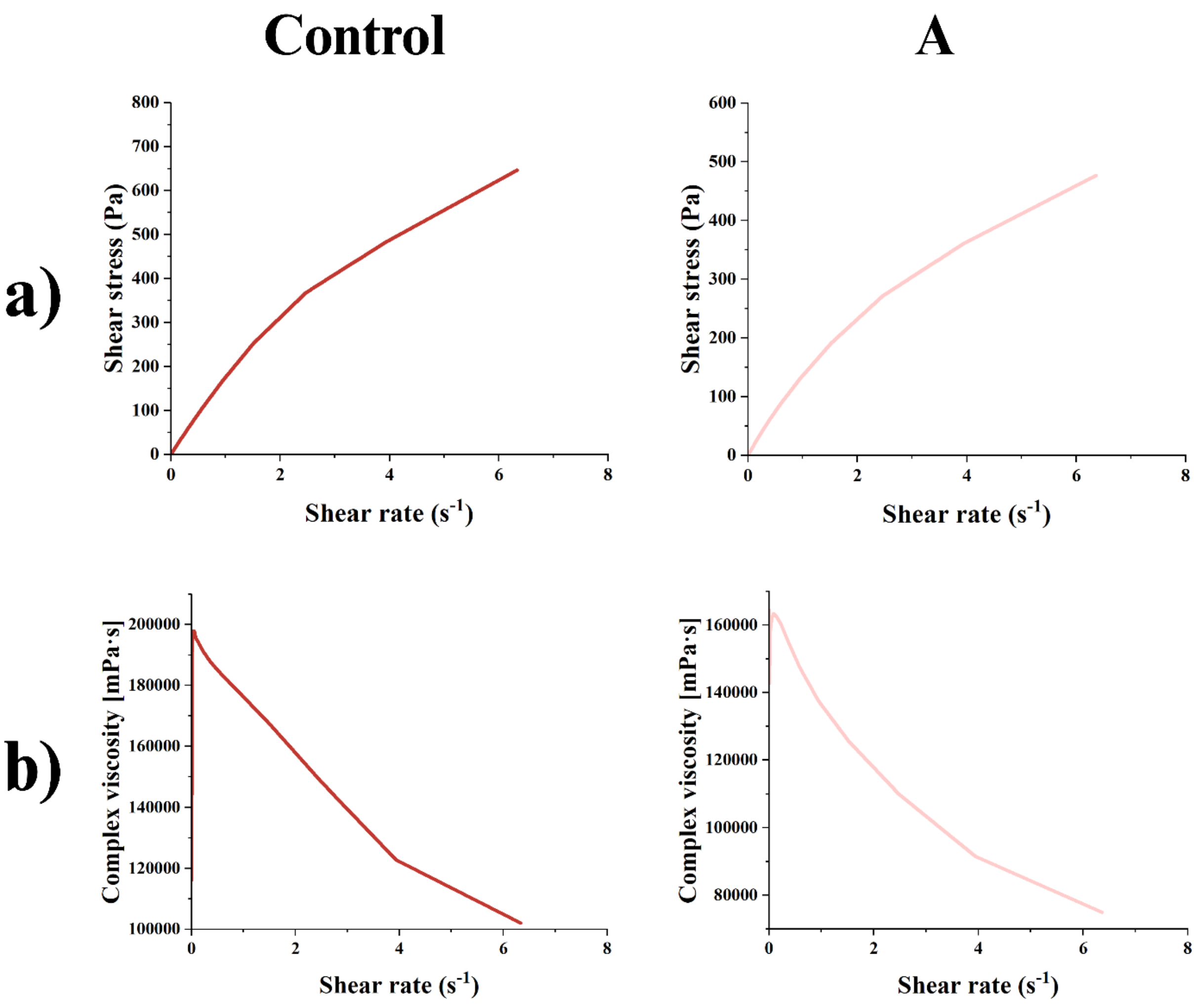

Figure S6: a) Representative flow curve (Shear stress vs. Shear rate), b) complex viscosity vs. shear rate of hydrogel Ct and A.

The flow curve (**Figure S6a**) shows a nonlinear increase in shear stress with shear rate, characteristic of a pseudoplastic behavior. This indicates that the hydrogel exhibits shear-thinning properties, where viscosity decreases under increasing shear, due to the partial alignment and reversible disruption of its internal polymeric network.

The complex viscosity decreased markedly with increasing shear rate (**Figure S6b**), indicating typical shear-thinning behavior. This pseudoplastic response arises from the reversible disruption and alignment of the polymeric chains within the hydrogel network under shear. Such behavior is characteristic of viscoelastic, cross-linked hydrogels and supports the presence of strong

intermolecular interactions contributing to the material's self-healing and structural recovery capabilities.

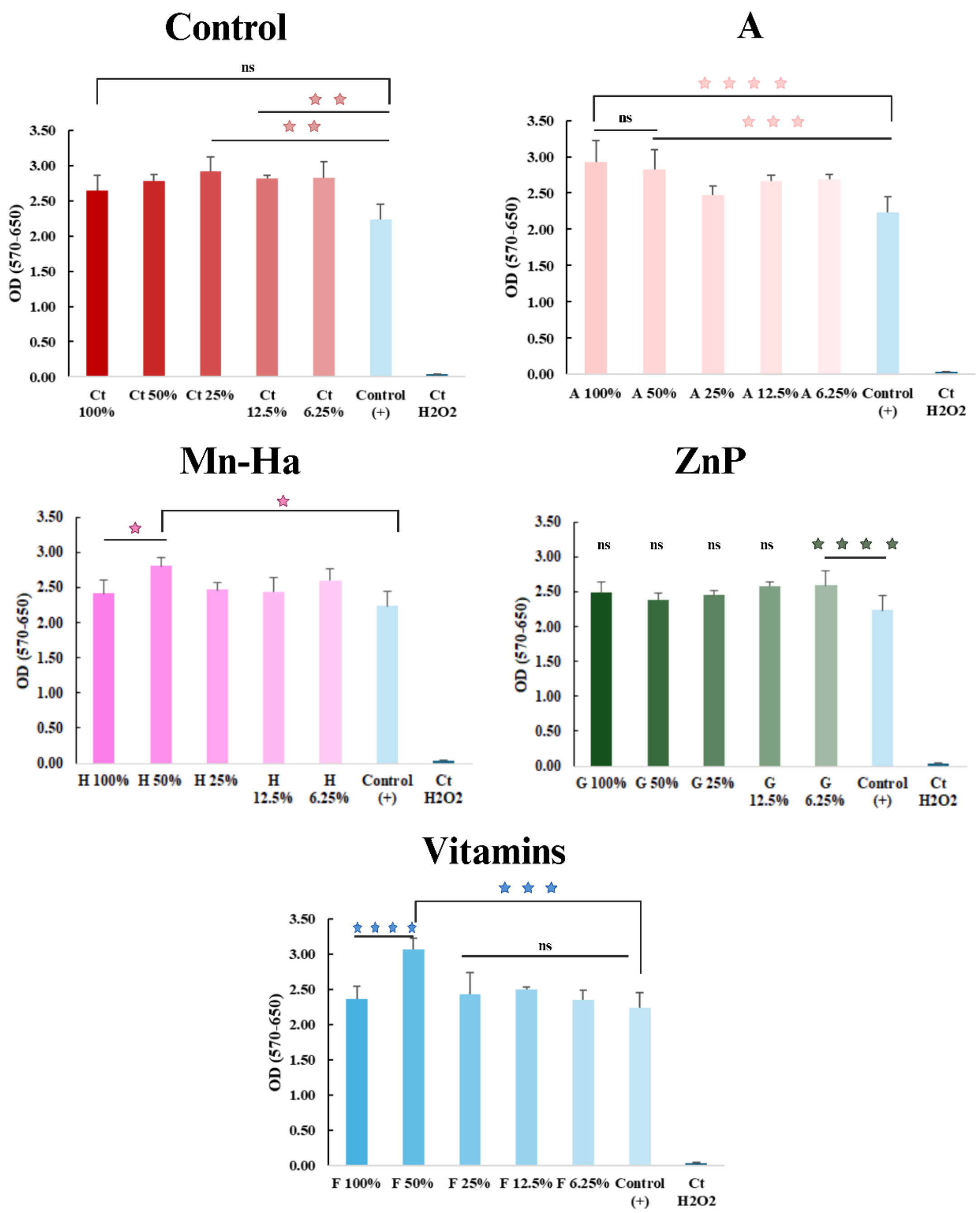

Figure S7: Cytotoxicity assays on MG-63 cells in experimental hydrogels for 1 day. The concentration of Ct, A, Ct+Mn-Ha, Ct+ZnP, and Ct+vitamins hydrogels were 100%, 50%, 25%, 12.5%, 6.25%. (★) Show a significant difference, $p < 0.05$, using one-way ANOVA followed by a post hoc Tukey test. No significant difference (ns). (*$p < 0.05$, **$p < 0.01$, ***$p < 0.001$). Data are presented as mean ± SD, n=4.

Table S1: Composition of hydrogels Control and A. *Solution of ZnP with a concentration of 2000 ppm.

	Materials	Control (g)	Control (wt%)	A (g)	A (wt%)
1	GG	0.232	1.16	0.232	1.00
2	CS	0.116	0.58	0.116	0.50
3	Gli	3.603	18.02	3.603	15.57
4	Glu	-	0.00	0.084	0.36
5	Con	-	0.00	0.084	0.36
6	Mn-Ha	-	0.00	0.025	0.11
7	Gel (4.28%w/v)	4.935	24.68	4.935	21.32
8	Col (1.4%w/v)	1.164	5.82	1.164	5.03
9	ZnP*	-	0.00	0.334	1.44
10	Ol	-	0.00	0.084	0.36
11	AH	-	0.00	0.286	1.24
12	Dp	-	0.00	0.572	2.47
13	ABA	-	0.00	0.835	3.61
14	Cal	-	0.00	0.535	2.31
15	Hamm	-	0.00	0.267	1.15
16	NaOH	0.194	0.97		0.00
17	Bx (4% w/v)	3.966	19.83	3.966	17.13
18	AT	0.116	0.58	0.116	0.50
19	Lec	-	0.00	0.084	0.36
20	VitA (2400 IU)	-	0.00	0.008	0.03
21	VitE (450 IU)	-	0.00	0.054	0.23
22	VitK2	-	0.00	0.07	0.30
23	VitD3 (125 IU)	-	0.00	0.025	0.11
24	PVA (10% w/v)	5.669	28.35	5.669	24.49
	Total	19.995	100%	23.148	100%

References

[1] B. Valdez-Salas, K. Guillén-Carvajal, E. Beltrán-Partida, J. Salvador-Carlos, M. Curiel-Álvarez, and N. Nedev, "Synthesis of stable zinc-phosphate micro/nanoparticles under acid and alkaline conditions," Jun. 30, 2025. DOI: 10.21203/rs.3.rs-6908597/v1.

[2] L. S. Villaseñor-Cerón *et al.*, "Biocompatibility analysis and chemical characterization of Mn-doped hydroxyapatite," *J Mater Sci Mater Med*, vol. 34, no. 8, p. 40, Jul. 2023, DOI: 10.1007/s10856-023-06744-0.

[3] B. Valdez-Salas *et al.*, "Structure-activity relationship of diameter controlled Ag@Cu nanoparticles in broad-spectrum antibacterial mechanism," *Materials Science and Engineering: C*, vol. 119, p. 111501, Feb. 2021, DOI: 10.1016/j.msec.2020.111501.

[4] S. J. Kashyap, R. Sankannavar, and G. M. Madhu, "Hydroxyapatite nanoparticles synthesized with a wide range of Ca/P molar ratios and their structural, optical, and dielectric characterization," *Journal of the Korean Ceramic Society*, vol. 59, no. 6, pp. 846–858, Nov. 2022, DOI: 10.1007/s43207-022-00225-w.
